# Neurodevelopmental disorders and gut-brain interactions: exploring the therapeutic potential of pycnogenol through microbial-metabolic-neural networks

**DOI:** 10.3389/fcimb.2025.1601888

**Published:** 2025-06-11

**Authors:** Ling Chen, Zhiqiang Li, Yuying Fan

**Affiliations:** Department of Pediatrics, Shengjing Hospital of China Medical University, Shenyang, Liaoning, China

**Keywords:** neurodevelopmental disorders, attention-deficit hyperactivity disorder, autism spectrum disorder, epilepsy, pycnogenol, gut-brain axis

## Abstract

Neurodevelopmental disorders (NDDs), characterized by cognitive impairments and behavioral abnormalities, represent a clinically diverse group of conditions typically emerging during childhood or adolescence. Major subtypes encompass autism spectrum disorder (ASD), attention-deficit hyperactivity disorder (ADHD), and epilepsy. The pathogenesis of these disorders involves multifactorial interactions between genetic susceptibility (*Shank3* mutations in ASD), environmental triggers (prenatal toxins), neurotransmitter dysregulation (dopamine (GA) and γ-aminobutyric acid (GABA) systems) and immune dysregulation. Growing research highlights the gut-brain axis disruption as a potential contributor to NDDs pathophysiology, though systematic evaluation of therapeutic approaches targeting this axis and related gastrointestinal comorbidities (GIDs) remains limited. This review comprehensively examines the pathological mechanisms underlying ADHD, ASD, and epilepsy, while analyzing the reciprocal relationship between gut-brain axis dysregulation and GID manifestations in NDDs. Notably, abnormal activation of key signaling pathways including NF-κB, MAPK and PI3K/AKT/mTOR is strongly associated with the pathogenesis of NDDs. We further propose pycnogenol (PYC), a polyphenol extract of pine bark, as a natural compound with multiple bioactivities such as anti-inflammatory and antioxidant, can directly or indirectly affect the function of the gut-brain axis by regulating the structure of the intestinal microbial community (increasing the abundance of *Akkermansia muciniphila* and butyric acid-producing bacteria) and its metabolites, providing a new strategy for the treatment of NDDs.

## 1 Introduction

Neurodevelopmental disorders (NDDs) are a complex set of disorders that occur in childhood due to a variety of genetic or acquired etiologies (Turner et al., 2021; Lukens and Eyo, 2022), primarily including attention-deficit hyperactivity disorder (ADHD), autism spectrum disorder (ASD), intellectual disability, tourette syndrome (TS) (Morris-Rosendahl and Crocq, 2020), and childhood epilepsy, which are characterized by abnormalities of development or dysfunction of the central nervous system that result in cognitive, emotional, sensory, and motor disorders. In particular, ADHD is characterized by inattention, hyperactivity, and impulsivity (Trebaticka and Durackova, 2015). ASD also is a classic NDDs, which is characterized by impaired social interactions, difficulties in verbal communication, and repetitive stereotyped behaviors (Lord et al., 2020). Some researchers have proposed that the neurobiological mechanisms of ASD include abnormal synaptic function, neurotransmitter imbalance, and neuroinflammation; and epilepsy, a widely observed neurological disorder, manifests through repeated episodes of aberrant electrical brain activity, potentially causing loss of consciousness and compromised motor control. Currently, the global prevalence of neurodevelopmental disorders is on the rise (Chen and Geschwind, 2022), estimates of the prevalence of ADHD range from 20.8 to 44.5%, TD from 1.8 to 17.7%, and ASD from 2.3 to 10.3% (Frances et al., 2022), as well as the need for long-term care that NDDs patients are often subjected to, posing a huge economic and psychological burden on families and society (Trebaticka and Durackova, 2015). In recent years, the potential of natural compounds in the treatment of neurodevelopmental disorders has gradually gained attention, among which the pine bark polyphenol extract, a natural compound extracted from the bark of the French coastal pine tree, which main component is proanthocyanidins, has become a hot research topic due to its antioxidant, anti-inflammatory and neuroprotective effects (Schoonees et al., 2012). Studies have shown that pycnogenol (PYC) is able to positively affect the nervous system by scavenging free radicals, reducing oxidative stress (OS), inhibiting inflammatory responses, and modulating neurotransmitter function (Belviranli and Okudan, 2015). For example, in ASD, PYC exerts antioxidant and anti-inflammatory properties that maybe help to alleviate behavioral problems and neuroinflammation (Nattagh-Eshtivani et al., 2022). In addition, PYC has shown potential anticonvulsant and neuroprotective effects in epilepsy treatment (Goel and Saxena, 2019). As a natural compound, PYC offers new ideas and possibilities for the treatment of neurodevelopmental disorders, but its safety and long-term efficacy need to be verified by more high-quality clinical trials.

Concurrently, numerous investigations into the microbiota-gut-brain axis reveal that the gut microbiota engages in dynamic interactions with the brain, collectively referred to as the “gut-brain axis.” On the one hand, gastrointestinal (GI) motility, secretion, and digestion are regulated by the central nervous system (CNS) (Cheng et al., 2019). On the other hand, dysbiosis of the gut flora is closely linked to various diseases of the CNS (Brescia and Rescigno, 2021). For example, children with ASD have significantly lower relative abundance and lower α-diversity of the *Prevotella*, *Coprococcus*, and the unclassified *Veillonellaceae* in the gut (Kang et al., 2013). In addition, dysbiosis of the gut flora increases the prevalence of ADHD (Cenit et al., 2017). All this suggests that the gut-brain axis could be used as an adjunctive therapeutic strategy for neurodevelopmental disorders. In this review, we review the pathological mechanisms of NDDs, the role of the gut-brain axis and the regulatory network of related signaling pathways, and explore the prospects of PYC as a multi-targeted intervention strategy in the treatment of NDDs.

## 2 Natural pine bark extract: pycnogenol

### 2.1 Anti-inflammatory effect

Inflammation represents a sophisticated immune mechanism, primarily serving as a protective reaction to harmful stimuli like pathogens, cellular damage, or toxic agents (Rafiyan et al., 2023), can be categorized as acute and chronic inflammation, acute inflammation initiated quickly and contributing to the removal of damaged cells and pathogens, which contributes to the health of the host (Alderton and Scanlon, 2021). However, when acute inflammation develops into chronic inflammation, it results in the development of an abnormal inflammatory response and a detrimental cycle of long-term tissue damage that can lead to diseases such as diabetes, neurological disorders and cancer (Langworth-Green et al., 2023; Nigam et al., 2023). PYC’s primary bioactive constituents, proanthocyanidins, comprise oligomeric/polymeric chains of epicatechin and catechin subunits, accompanied by complementary phytochemicals like flavonoid derivatives, polyphenol monomers, and glycosylated cinnamic/phenolic acids (Rohdewald, 2018). These compounds demonstrate broad therapeutic potential against chronic inflammatory pathologies. Mechanistically, PYC suppresses macrophage-derived pro-inflammatory cytokine secretion (Nattagh-Eshtivani et al., 2022) while upregulating TReg cell-associated markers (Foxp3 and interleukin-10 (IL-10)) (Fan et al., 2015). Experimental evidence from Liu et al. revealed PYC’s capacity to attenuate lipopolysaccharide (LPS)-induced microglial inflammation by suppressing nitric oxide (NO), IL-6, and IL-1β production (Liu et al., 2024). Research indicates that lipopolysaccharide (LPS) triggers microglial inflammatory responses through activator protein-1 (AP-1) and nuclear factor kappa-B (NF-κB) pathways. LPS stimulation in microglia increases nitric oxide (NO) and pro-inflammatory cytokines IL-6 and IL-1β, whose levels correlate positively with AP-1 and NF-κB expression. Notably, PYC inhibits these transcription factors’ activity and attenuates pro-inflammatory mediator production (Fan et al., 2015), corroborating findings from Liu et al. Microglia, the resident immune cells of the CNS, are activated in processes tightly linked to neuroinflammation and essential for normal nervous system development (Lei et al., 2014).

### 2.2 Antioxidant effects

oxidative stress occurs when the equilibrium between oxidants and antioxidants is disrupted, leading to overproduction of reactive oxygen species (ROS) and free radicals. These disturbances in homeostasis are associated with numerous diseases, such as cardiovascular conditions, tumors, and neurological disorders (van der Pol et al., 2019). Research suggests that PYC inhibits the expression of cell adhesion molecules, cyclooxygenase (COX), lipoxygenase (Peng et al., 2012), NO, and inducible nitric oxide synthase (iNOS) (Uhlenhut and Hogger, 2012). This is due to the fact that the main active ingredients of PYC include proanthocyanidins, catechins, phenolic acids and flavonoids, which are effective in scavenging free radicals in the body, thus reducing cellular damage caused by OS (Packer et al., 1999). Studies have shown that PYC not only scavenges free radicals directly, but also protects the cellular antioxidant system and enhances the antioxidant defense of cells (Kim B. et al., 2020). Cossin et al. used ESR spectroscopy to study the retention time of ascorbate radicals in the ascorbate-ascorbate oxidase system and found that the retention time was prolonged after PYC treatment (Cossins et al., 1998). In addition, the protective effect of PYC on the nervous system is one of its important functions. It has been proposed that amyloid beta peptide (Aβ) induced a large amount of ROS production by PC12 of pheochromocytoma cells leading to apoptosis (Cheng et al., 2017), whereas PYC treatment inhibited the release of ROS, which ultimately protected the neuronal cells from A beta-induced apoptosis, and improved the improvement of memory and cognitive functions (Peng et al., 2002). PYC is able to enhance synaptic plasticity by promoting the expression of brain-derived neurotrophic factor (BDNF) (Kebir and Joober, 2011), an important neurotrophic factor that promotes neuronal growth, differentiation and synapse formation. Studies have shown that PYC can significantly increase BDNF levels, resulting in improved synaptic function and learning memory (Luzzi et al., 2011). Additionally, another clinical study showed that the ingestion of 1 mg/kg of PYC for four weeks in pediatric patients with ADHD resulted in 18.3% improvement in hyperactivity and 14.4% improvement in attention (Weichmann and Rohdewald, 2024), and DNA damage, as assessed by measuring the levels of 8-oxo-7,8-dihydroguanine (8-oxoG), found that oxidative DNA damage in pediatric patients with ADHD after ingestion of PYC significantly decreased, as well as increased total antioxidant status and improved glutathione (GSH) levels in pediatric patients with ADHD after PYC intake (Weichmann and Rohdewald, 2024). Treatment of ADHD in children with PYC normalized catecholamine concentrations, leading to a reduction in OS and alleviation of ADHD (Sinn, 2008). These studies suggest that PYC can be a strong candidate for the treatment of neurodevelopmental disorders by exerting its antioxidant effects (**Table 1**).

**Table 1:** Treatment effects of PYC on ADHD, ASD and epilepsy.

Diseases	Sample volume	PYC dose	Duration	Effects	Reference
ADHD	61 children	1 mg/kg/d	4 weeks	↓ Hyperactivity and inattention; ↓ plasma OS markers	(Trebaticka et al., 2006)
ADHD	24 adults	1 mg/kg/d (Four doses)	3 weeks	↑ Executive functioning	(Tenenbaum et al., 2002)
ADHD	50 children	1 mg/kg/d	12 weeks	↓ GSSG; ↑ GSH; ↓ TAS	(Dvorakova et al., 2006)
ADHD	144 children	1 mg/kg/d	10 weeks	↓ ADHD-RS scores; Improve ADHD and focus	(Verlaet et al., 2017)
ADHD	57 children	1 mg/kg/d	4 weeks	↓ Catecholamine levels, OS and hyperactivity	(Dvorakova et al., 2007)
ASD-induced lung inflammation	10 mice (n=5)	15 mg/kg and 30 mg/kg	6 days	↓ Inflammatory cell infiltration; ↓ IL-1β, IL-6, TNF-α; Inhibited MMP-9 expression	(Pak et al., 2024)
Mice model of pentylenetetrazole-induced epilepsy	36 mice (n=6)	50 mg/kg and 100 mg/kg	2 weeks	Prolonged seizure latency; ↓ Seizure duration and frequency; ↓ TBARS; ↑SOD, CAT and GSH	(Goel and Saxena, 2019)

↓ Expression downward, ↑ Expression upward.

### 2.3 PYC metabolism and gut microbiota

**Figure 1:**
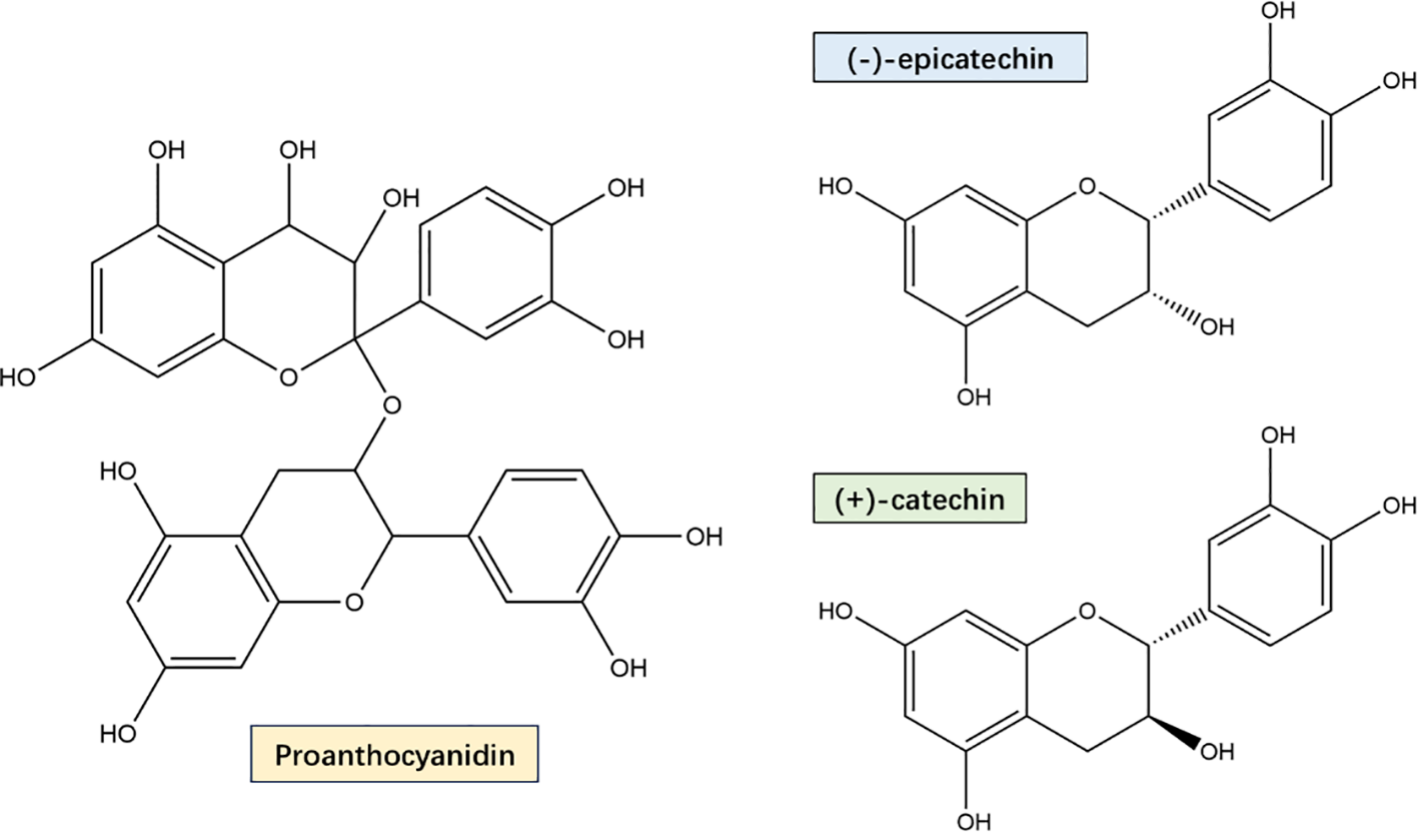
PYC main components proanthocyanidin, catechins and epicatechins Structural formula. PYC’s main components are a mixture of monomers, oligomers, and polymers, and its monomers are mainly catechin and epicatechin. Oligomers and polymers are collectively known as proanthocyanidin, which are bioflavonoids with a special molecular structure, and are now internationally recognized as the most effective natural antioxidants for scavenging free radicals in the human body.

PYC is derived from French coastal pine bark extract and is standardized to contain 70 ± 5% proanthocyanidins, consisting of catechin and epicatechin polymers with different chain lengths, as well as low molecular weight compounds containing paclitaxel, vanillic acid, gallic acid, cinnamic acid, ferulic acid, and caffeic acid (**Figure 1**) (D’Andrea, 2010). These low molecular weight compounds are able to be absorbed in the small intestine and the oligomers and polymers are metabolized by microorganisms upon arrival in the large intestine. After reaching the large intestine, they are metabolized by microorganisms (Schantz et al., 2010). A pharmacokinetic study showed that plasma samples assayed from healthy volunteers given 200 mg (multiple intakes over 5 days) and 300 mg (single-dose intake) of PYC, tested 4 h after the last intake and 14 h after the single-dose intake, revealed that catechins, paclitaxel, caffeic acid and ferulic acid, as well as the intestinal microbial the metabolite 5-(3′,4′-dihydroxyphenyl)-γ-valerolactone content was significantly increased (Grimm et al., 2006). In addition, the presence of oligomers and polymeric proanthocyanidins in the large intestine promotes the proliferation of a variety of beneficial bacteria with health-protective effects on the host. For example, *Akkermansia muciniphila* and butyrate-producing bacteria showed increased colonisation while inhibiting LPS-producing bacteria (Niwano et al., 2022). Proanthocyanidins have been reported to increase the abundance of gut microorganisms such as *Bacteroidota*, *scillospiraceae*, *Muribaculaceae* and *Desulfovibrionaceae*, as well as activate short-chain fatty acids (SCFAs) receptors in the colon (Sun et al., 2024). Another study claimed that catechin intervention induced an increase in the ratio of Bacteroidetes to Firmicutes, as well as a decrease in the relative abundance of *Lactobacillus plantarum* and *Acetobacter pomorum (* Perez-Burillo et al., 2021). Along with the promotion of beneficial bacteria and the inhibition of harmful bacteria by PYC, which can contribute to the formation and differentiation of intestinal immune cells and regulate the release of inflammatory factors, thus affecting brain activity and function (Wu et al., 2025). And the fact that SCFAs can stimulate the vagus nerve or indirectly regulate host metabolism and cognition through immune-neuroendocrine mechanisms (Doifode et al., 2021), these studies have demonstrated that PYC has a protective effect on the nervous system through the gut-brain axis for neuroprotective effects. Furthermore, since PYC is primarily composed of phenolic compounds, these compounds undergo biotransformation upon ingestion. Specifically, they are metabolized by microbial enzymes in the colon, resulting in the production of smaller bioavailable molecules. These metabolites are subsequently absorbed by the colon into the bloodstream and transported to various tissues and organs (Rohdewald, 2002). During the metabolism of PYC, intestinal microorganisms play a critical role in the catabolism and transformation of phenolic compounds, leading to the formation of small molecule metabolites with antioxidant and anti-inflammatory bioactivities. By analyzing the serum metabolites of human volunteers after PYC ingestion, it was observed that the metabolites 5-(3′,4′-dihydroxyphenyl)-γ-valerolactone and 5-(3′-methoxy-,4′-hydroxyphenyl)-γ-valerolactone were retained in blood cells and exerted beneficial effects on host health (Bayer and Hogger, 2024).

## 3 Pathogenesis of neurodevelopmental disorders

ADHD is a NDDs that usually begins in early childhood and is persistent (Cortese and Coghill, 2018), characterized by inattention, impulsivity, hyperactivity, and poor control over self-behavior. Globally, the prevalence of ADHD in children and adolescents in recent years has been about 8.0%, with the prevalence in boys (10%) being twice as high as in girls (5%) (Ayano et al., 2023). The majority of ADHD patients have normal intelligence but often have cognitive impairment and learning difficulties, and the development of ADHD involves genetic (Laria et al., 2021), environmental (Faraone and Larsson, 2019), dietary, neurotransmitter, and brain function abnormalities. Studies of twins and adopted children have shown that ADHD is highly genetically inherited (60%-90%) (Faraone et al., 2015). Although the specific pathogenesis of ADHD has not yet been elucidated, neurotransmitter abnormalities (dopamine (DA), norepinephrine (NE), 5-hydroxytryptophan (5-HT)) as well as increased OS and neuroinflammation in patients with ADHD have been the focus of most research (Curatolo et al., 2009).

ASD is a heterogeneous lifelong NDDs primarily characterized by social difficulties, communication difficulties and repetitive behaviors and interests (Hirota and King, 2023). The prevalence of ASD is reported to be steadily increasing worldwide, reaching 1.85% in 2016 alone, with a 4.3 times more common in boys than girls (Maenner et al., 2020), often accompanied by symptoms such as epilepsy, anxiety, depression and ADHD (Yang et al., 2022). The study suggested that genetic and environmental factors early in development play a crucial role in the aetiology of autism (Maenner et al., 2020). The researchers found that ASD runs predominantly in families, with a heritability of 83%, especially in twins, where the proportion of phenotypic variation due to genetic factors is estimated to be as high as 90%, suggesting that genetic factors may explain most of the etiology of ASD (Sandin et al., 2017). In addition, chemical exposures, infections, inflammation and dysfunctional immune responses during early embryonic development also increase the prevalence of ASD (Li et al., 2021). Furthermore, we summarize and highlight the mechanistic advances in synaptic function and pathway abnormalities, gut-brain axis and immune response dysregulation in ASD patients to provide a theoretical basis for the next step in precision therapy.

Epilepsy, the most prevalent neurological disorder, affects approximately 0.5% to 2% of the general population and is characterized by recurrent epileptic seizures (Christodoulou et al., 2018). These seizures can present with a range of clinical manifestations, including loss of consciousness, limb convulsions, foaming at the mouth, and incontinence. Notably, epidemiological data indicate that approximately two-thirds of epilepsy cases have their onset during childhood or adolescence (Christodoulou et al., 2018). The pathogenesis of epilepsy is highly complex and multifactorial, involving a combination of mechanisms such as abnormal neuronal discharges, imbalances in neurotransmitter systems, dysfunction of ion channels, dysregulation of neuroglial cell activity, genetic predispositions, immune-mediated processes, mitochondrial dysfunction, and the modulatory influence of gut microbiota (Chakraborty et al., 2022). In recent years, advances in genomic technologies, such as whole exome sequencing (Palmer et al., 2018), have unveiled the intricate genetic interplay between epilepsy and neurodevelopmental disorders. For instance, Takata et al (Takata et al., 2019). demonstrated that individual-specific deleterious ultra-rare variants (dURVs) are significantly enriched in patients with epileptic encephalopathy (EE) and developmental and epileptic encephalopathy (DEE). These variants were identified not only within known EE/DEE-associated genes but also in genes not previously linked to these conditions (Takata et al., 2019). DEE represents a distinct epilepsy subtype characterized by the co-occurrence of seizures and neurodevelopmental impairments, wherein seizures may exacerbate neurodevelopmental deficits, and conversely, neurodevelopmental disorders may heighten seizure susceptibility (Guerrini et al., 2023). In the following sections, we will provide a detailed exploration of epilepsy-related pathological mechanisms, with a focus on abnormal neuronal excitability and mitochondrial dysfunction.

### 3.1 Neurotransmitter abnormalities

Neurotransmitters play an important role in the regulation of neuronal activity, cognitive function and behavioral control. The development of ADHD is closely linked to abnormal levels of neurotransmitters such as DA (Faltraco et al., 2021), NE and 5-HT (Bidwell et al., 2011; Daws and Gould, 2011). Modulation of attention, energy levels, and emotional control relies heavily on these neurotransmitters. Notably, ADHD-diagnosed children display distinct DA and NE level profiles relative to their neurotypical counterparts (Gul et al., 2022), and this difference leads to impaired messaging and regulation. In patients with ADHD, dysfunction of the DA system is mainly characterized by reduced levels of dopamine or obstruction of its delivery processes (Li et al., 2017). For example, positron emission tomography studies have found that ADHD patients have an increased density of dopamine transporters in brain regions such as the prefrontal cortex and the striatum (Koirala et al., 2024), which allows for a too-rapid reuptake of dopamine, thereby reducing the effective concentration of dopamine in the synaptic gap, ultimately leading to distraction and impulsive behavior (Koirala et al., 2024). In addition, animal experiments have shown that activation of dopamine D_2_ receptors significantly enhances working memory and attention, but the density of dopamine D_2_ receptors is significantly reduced in the prefrontal cortex of ADHD patients (Vazquez et al., 2022), which further contributes to cognitive dysfunction and behavioral inhibition. Genetically, the genetic predisposition to ADHD is further supported by the findings of dopamine receptor D_4_ and dopamine receptor D_5_ as ADHD susceptibility genes. NE is another important neurotransmitter closely related to ADHD and is mainly involved in regulating functions such as alertness, attention and mood (Vazquez et al., 2022). Research suggests that the function of the norepinephrine transporter may be enhanced in people with ADHD, leading to increased reuptake of norepinephrine, which reduces its concentration in the synaptic gap (Bonisch and Bruss, 2006). Decreased norepinephrine impairs the brain’s ability to regulate attention and alertness, resulting in inattention and hyperactive behavior.

Epilepsy is fundamentally characterized by abnormal neuronal activity, primarily driven by an imbalance between excitatory and inhibitory neurotransmission, which leads to the pathological over-synchronization of neuronal networks (Follwaczny et al., 2017). A key mechanism underlying this imbalance involves the excessive release of glutamate, the brain’s principal excitatory neurotransmitter, and/or insufficient levels of γ-aminobutyric acid (GABA), its major inhibitory counterpart (Akyuz et al., 2021). GABA, the primary inhibitory neurotransmitter in the brain, is predominantly localized within short-axoned interneurons (Treiman, 2001). These interneurons form synaptic connections with neuronal cell bodies and proximal axons, playing a critical role in maintaining inhibitory tone and counterbalancing excessive neuronal excitation to ensure neural circuit stability (Treiman, 2001). Recent studies have demonstrated that transplantation of GABAergic interneuron precursor cells can enhance endogenous GABA signaling, offering a promising therapeutic strategy for seizure control (Arshad et al., 2023). Additionally, many antiepileptic drugs exert their anticonvulsant effects by modulating GABAergic transmission, such as through the potentiation of GABA receptor activity or inhibition of GABA reuptake (Loscher, 2021). Additionally, the voltage-gated sodium ion (Na_V_) α subunit 1 (*SCN1A*), which encodes the Na_V_1.1 subunit, is predominantly expressed in GABAergic neurons (Parihar and Ganesh, 2013). Extensive research has identified *SCN1A* mutations as one of the most frequently implicated genetic alterations in epilepsy (Parihar and Ganesh, 2013). Experimental studies using *SCN1A* knockout mice have demonstrated that the loss of Na_V_1.1 function selectively reduces Na^+^ current density in inhibitory interneurons, while sparing excitatory pyramidal neurons (Chakraborty et al., 2022). This interneuron-specific impairment disrupts inhibitory neurotransmission, leading to neuronal hyperexcitability and the development of epilepsy. Meanwhile, *SCN1B* encodes Na_V_β1, which shares similar clinical features with *SCN1A (* Brackenbury and Isom, 2011), suggesting that the mechanism of pathogenesis of *SCN1B* mutations may involve impaired Na_V_1.1 function, ultimately leading to abnormal Na^+^ channels (Wei et al., 2017). Notably, exome and targeted sequencing revealed that K^+^ channel dysfunction may also lead to ineffective reduction of excitability, which may predispose to epilepsy. Voltage-gated potassium (K_V_) channels have an essential function in the modulation of electrical excitability in neuronal systems (Zheng and Chen, 2024). Research has shown that mutant K_V_ channels are associated with epilepsy (Zheng and Chen, 2024), indicating that abnormal ion channel function is closely linked to seizures.

### 3.2 Oxidative stress and neuroinflammation

Recent evidence suggests that ADHD is strongly associated with redox imbalance. OS in the brain causes damage to neuronal integrity due to the high susceptibility of polyunsaturated fatty acids to oxidation and ROS production, which may also lead to activation of astrocytes and microglia. Some of these enzymes are catalase (CAT), superoxide dismutase and glutathione peroxidase (GPx), which are accepted biomarkers of OS. In a study of 35 ADHD patients and 35 healthy controls, plasma GPx levels were found to be significantly lower in the ADHD patients than in the healthy group (Ceylan et al., 2010). The mean serum levels of TAC, GSH and CAT were significantly lower in the ADHD patients than in the healthy group (Visternicu et al., 2024). Recently, it has been reported that pediatric patients with ADHD have higher levels of OS, particularly malondialdehyde (Bulut et al., 2007), a breakdown product of the main chain reaction leading to polyunsaturated fatty acid oxidation used as a marker of OS (Corona, 2020). Meanwhile, when redox imbalance occurs, the escape of high levels of ROS activates the massive release of pro-inflammatory chemokines and cytokines, which dysregulates the immune response and ultimately leads to neuroinflammation (Weng et al., 2018). In one study, elevated levels of the cerebrospinal fluid pro-inflammatory cytokine TNF-β and low levels of the anti-inflammatory cytokine IL-4 were observed in patients with ADHD (Mittleman et al., 1997). Gustafsson et al. suggested that levels of IL-6, tumor necrosis factor-α (TNF-α), and monocyte chemotactic protein-1 may be markers of ADHD risk (Gustafsson et al., 2020). Prenatal exposure to inflammation has been reported to potentially disrupt brain development, causing structural changes in gray matter volume and brain regions (prefrontal cortex, anterior cingulate cortex, and corpus callosum), resulting in failure of permanent neural circuits to mature, or neuroendocrine changes that increase the risk of ADHD in children (Buske-Kirschbaum et al., 2013). Animal studies have shown that adult mice prenatally exposed to polycytidylic acid (poly(I:C)) have a reduction in total brain volume (da Silveira et al., 2017) similar to the reduction in gray matter volume in children with ADHD (Castellanos et al., 2002). Thus, OS and neuroinflammation are coexisting and interrelated mechanisms (Solleiro-Villavicencio and Rivas-Arancibia, 2018).

A growing body of research suggests that the pathogenesis of ASD is related to the accumulation of oxidative products and disturbances in antioxidant metabolism. When oxidative stress occurs in the endothelium of children with autism, the blood-brain barrier may be impaired, leading to differential diffusion and transport (Ellwanger et al., 2016). Studies have shown that compared to healthy controls, blood levels of plasma malondialdehyde, serum malondialdehyde, RBC superoxide dismutase, plasma reduced glutathione, and Plasma glutathione, which are markers of oxidative stress, were reduced or increased in varying degrees (Geier et al., 2009; Hu et al., 2020). Usui N. et al. found, by analyzing lipid metabolomics, that 48 metabolites in patients with ASD were enriched mainly in lipid biosynthesis and metabolism, oxidative stress and Synaptic function-related targets (Usui et al., 2020). Meanwhile, coenzyme Q10, a mitochondrial antioxidant cofactor that crosses the BBB, was shown to improve communication in children with ASD by administering its reduced form (ubiquinol) to children with ASD (Gvozdjakova et al., 2014). In addition, the direct administration of coenzyme Q10 had a significant mitigating effect on the symptoms and oxidative stress in ASD (Mousavinejad et al., 2018).

Simultaneously oxidative stress is a key factor in the onset and development of epilepsy. The production and accumulation of free radicals leads to cell damage and neuronal death, which can trigger epilepsy (Waldbaum et al., 2010). Studies have proposed that antioxidants can reduce seizure frequency and severity in animal models (Aguiar et al., 2012). Whereas mitochondria serve as a major source of ROS, mitochondrial dysfunction leads to overproduction of ROS, further exacerbating OS (Aguiar et al., 2012). Mutations in mitochondrial DNA have been found in hereditary epilepsy (myoclonic epilepsy with ragged red fibers) and mitochondrial encephalopathies that can directly affect the activity of the electron transport chain (Pearson-Smith and Patel, 2017), as well as the fact that most mouse models deficient in SOD2 suffer from mitochondrial dysfunction, resulting in increased ROS, decreased ATP, and ataxia (Liang et al., 2012). These studies suggest a vicious cycle of OS and mitochondrial dysfunction in epilepsy that can contribute to lower seizure thresholds and increased severity.

### 3.3 Synaptic function and pathway abnormalities

It is proposed that most ASD risk genes encode proteins that play an important role in synaptic function, such as neurexins (NRXNs, such as NRXN1, NRXN2, NRXN3) (Costales and Kolevzon, 2015), Neuroligins and *Shank3 (* Lyons-Warren et al., 2022). For example, mutations in the NRXN1 gene, which encodes a synaptic adhesion molecule and a presynaptic neuronal connexin that plays an important role in synaptic adhesion (Li and Pozzo-Miller, 2020), differentiation and maturation, have also been found to be strongly associated with the development of ASD. And animal studies showed that inhibitory synapses were reduced in the brainstem and cortex of all three NRXN knockout or double knockout KO animals. Several brain regions are reduced in volume in Nlgn^3R451C^ KI mice, which carry Nlgn^3R451C^ mutations associated with ASD patients (Ellegood et al., 2011), and show reduced hippocampal neurotransmission mediated by AMP-activated protein kinase (AMPK) receptors (Chanda et al., 2016). *Shank3* is a postsynaptic density (PSD) protein that has an essential function in targeting neurons (Wang L. et al., 2023), anchoring and dynamic regulation of synaptic localization of neurotransmitter receptors and signaling molecules (Zhou et al., 2016). Moreover, molecular, biochemical and behavioral abnormalities associated with autism phenotypes have been observed in various Wnt pathway-associated knockout mouse models (Grayton et al., 2013; Durak et al., 2016). As a negative regulator of autism, CHD8 can participate in the classical Wnt signaling pathway by binding directly to β-catenin or being recruited to the promoter region of β-catenin responsive genes (Durak et al., 2016). In addition, β-catenin is closely linked to the Wnt signaling pathway and regulates normal brain development along with phosphatase and tensin homolog (PTEN) (Kwan et al., 2016). Furthermore, it has been shown that the PI3K/AKT/mTOR is highly correlated with ASD and is mainly involved in regulating synaptogenesis (Modi and Sahin, 2020), cortico-genesis and related neuronal processes (Gilbert and Man, 2017). AKT/mTOR is a biological substrate for autism, and mTOR is involved in synaptic plasticity, neuronal development and memory storage in the CNS (Trovato et al., 2020). Protein expression and phosphorylation of mTOR and its downstream signaling pathway components were found to be significantly reduced in autistic individuals (Nicolini et al., 2015).

### 3.4 Gut microbiology and immune dysregulation

Patients with ASD frequently exhibit comorbid GI dysfunction, a phenomenon that may be mechanistically linked to the complex interplay between the GI system and neuroendocrine/neuroimmune systems (Vuong and Hsiao, 2017; Hughes et al., 2018). This observation aligns with the growing body of research on the gut-brain axis, which has gained significant attention in recent years for its role in bidirectional communication between the gut and the central nervous system (Vuong and Hsiao, 2017). The gut microbiota, defined as the diverse community of microorganisms residing in the human gastrointestinal tract, is increasingly recognized as a key mediator in this cross-system interaction. Given that ASD typically manifests during childhood, a critical period when the infant gut microbiota is shaped by oligosaccharide-rich breast milk (Rodriguez et al., 2015), the healthy infant gut microbiota is predominantly colonized by beneficial genera such as *Bifidobacterium* and *Lactobacillus (* Human Microbiome Project, 2012). However, studies have identified significant dysbiosis in children with ASD, characterized by elevated levels of *Desulfovibrio*, a sulfate-reducing bacterium whose abundance correlates with autism severity (Tomova et al., 2015). Notably, its primary metabolic byproduct, hydrogen sulfide, exhibits cytotoxic effects on colonic epithelial cells and contributes to GI inflammation, distinguishing ASD patients from healthy controls (Carbonero et al., 2012). Comparative analyses of gut microbiomes between ASD patients and their neurotypical siblings have revealed substantial alterations in microbial composition. Specifically, significant reductions were observed in the relative abundances of *Faecalibacterium prausnitzii* (a key butyrate producer with anti-inflammatory properties), *Prevotella copri*, *Bacteroides fragilis*, and *Akkermansia muciniphila (* Retuerto et al., 2024), alongside a decreased ratio of Bacteroidetes and Firmicutes (Tomova et al., 2015). Furthermore, ASD-associated microbiomes exhibited a marked depletion of genera involved in carbohydrate degradation and fermentation, including *Prevotella*, *Coprococcus*, and unclassified *Veillonellaceae*, suggesting impaired microbial metabolic functionality (Kang et al., 2013). In contrast, the gut microbiota of patients with ADHD and epilepsy is unclear. One study found that the relative abundance of *Bifidobacterium* was lower in pediatric patients with ADHD (Partty et al., 2015). In a study by B. Jakobi et al. it was shown that in adult patients with ADHD the relative abundance of *Clostridia_UCG_014* and *Eubacterium_xylanophilum_group* was decreased, whereas the relative abundance of *Eisenbergiella* and *Ruminococcus_torques_group* was increased (Jakobi et al., 2024). *Ruminococcus_torques_group* was previously reported to be enriched in pediatric patients with ASD and to play a significant role in gut microbiota function and pro-inflammatory responses (Jakobi et al., 2024). It has also been suggested that the transfer of gut microbes from ADHD patients to healthy mice resulted in altered flora structure (Tengeler et al., 2020). In addition, one study analyzed the causal relationship between gut microbiota and epilepsy and found that certain specific gut flora (*Betaproteobacteria*, *Veillonellaceae*, and *Burkholderiales*) were associated with an increased risk of epilepsy (Zeng et al., 2023; Qiu et al., 2024).

Concurrently, immune dysregulation has been identified as a common feature in individuals with ASD, with studies demonstrating a strong association between immune dysfunction and the severity of behavioral symptoms (Hughes et al., 2023). Notably, children with ASD exhibit elevated levels of circulating innate immune cells, further underscoring the interplay between systemic inflammation and neurodevelopmental outcomes in this population (Tural Hesapcioglu et al., 2017; Robinson-Agramonte et al., 2022). Additionally, Enstrom et al. demonstrated dysregulation of monocyte activation in patients with ASD. Their findings revealed that Toll-like receptor 2 activation in peripheral CD14^+^ monocytes isolated from ASD patients led to significantly elevated expression of TNF-α, IL-1β, and IL-6 (Kim YS. et al., 2020). Notably, LPS-induced TLR4 activation also resulted in increased IL-1β expression. Importantly, the study established a positive correlation between elevated IL-1β levels and the severity of ASD-related behavioral manifestations (Emanuele et al., 2010). Elevated pro-inflammatory cytokine responses, particularly interferon-γ and TNF-α, have been identified as significant contributors to the heightened susceptibility of autistic children to GI inflammation and the exacerbation of behavioral symptoms (Hughes et al., 2023). Studies have demonstrated that these cytokines are produced in response to common dietary proteins in autistic children, highlighting a potential link between dietary triggers and systemic inflammation (Schwartzer et al., 2017). Importantly, these pro-inflammatory cytokines can either cross the blood-brain barrier (BBB) directly or stimulate brain endothelial cells, disrupting the local immune milieu within the central nervous system (Robinson-Agramonte et al., 2022). This immune dysregulation fosters a dysfunctional brain environment, which is thought to underlie core behavioral manifestations of ASD, including social dysfunction and repetitive behaviors.

## 4 Gut microbiota and neurodevelopmental disorders

### 4.1 Gut microbes linked to neurodevelopment

The gut microbiota critically regulates brain activity and behavioral processes, a mechanism largely mediated by the gut-brain axis, a bidirectional signaling pathway connecting the gastrointestinal tract and the central nervous system, as evidenced by extensive scientific inquiry (Davias et al., 2025). Experimental studies utilizing animal models have revealed that both germ-free (GF) mice and antibiotic-treated, microbiota-depleted mice exhibit profound neurophysiological and behavioral alterations compared to control groups (Vuong et al., 2020). These changes manifest as reduced social interaction, impaired cognitive performance, and heightened anxiety-like behaviors. Conversely, when neonatal animals with depleted microbiota undergo gut flora diversification or complete microbial reconstitution, significant improvements are observed in brain development and behavioral abnormalities previously seen in GF mice. Epidemiological case-control studies have further demonstrated that children with neurodevelopmental disorders, exhibit significant alterations in gut microbiota composition (Dan et al., 2020). These findings suggest a potential link between gut microbial dysbiosis and the pathogenesis of neurodevelopmental disorders. In addition, Hsiao et al (Hsiao et al., 2013). showed that the probiotic *Bacteroides fragilis* was able to remodel the gut microbiome and improve social, repetitive and anxiety-like behaviors in mice.

Notably, gut microbial metabolites are regulating host neurodevelopment through multiple mechanisms. Studies have shown that specific genera including *Alloprevotella*, *Paraprevotella* and *Ruminococcus* exert neuroprotective effects by producing SCFAs that inhibit microglial cell activation and attenuate neuroinflammation (Deng et al., 2025). The metabolites of these SCFAs have been demonstrated to enhance host cognitive function, improve learning and memory, and promote emotional stability (Deng et al., 2025). SCFAs are essential for the maintenance of immune homeostasis in the brain and the regulation of neuroinflammatory processes (Hoogland et al., 2015). In particular, butyrate regulates gene expression by inhibiting histone deacetylase (HDAC) activity (Ullah et al., 2024), thereby promoting neuronal plasticity and supporting cognitive function (Selkrig et al., 2014). These findings emphasize the importance of gut-derived metabolites in shaping brain health and function. Furthermore, integrated multi-omics analyses have revealed significant differences in the levels of specific neurotransmitter precursor metabolites and neurotransmitter metabolites between individuals with ASD and neurotypical controls (Dan et al., 2020). Notably, a substantial body of evidence indicates that elevated blood levels of 5-HT are commonly observed in children with ASD, a phenomenon potentially linked to gut microbiota-mediated regulation of tryptophan metabolism (Chen R. et al., 2017). Specific gut microbial species, including *Lactobacillus rhamnosus*(*L. rhamnosus*)and *Clostridium butyricum*, have been shown to modulate host mood and neuronal excitability through their influence on the secretion of key neurotransmitters and neuromodulators, such as glucagon-like peptide-1, 5-HT, and GABA (Dicks, 2022). In a recent preclinical study, administration of the probiotic *L. rhamnosus* was found to enhance cognitive function in mice (Bravo et al., 2011). This effect was attributed to the strain’s ability to regionally modulate GABA receptor expression in the brain, leading to reduced anxiety-like behaviors and improved memory performance (Bravo et al., 2011). These findings not only highlight the intricate interplay between gut microbiota and brain function but also open new avenues for exploring microbiota-based therapeutic strategies in neuropsychiatric disorders.

### 4.2 Gut microbiome dysbiosis and ADHD, ASD and epilepsy

#### 4.2.1 Altering the microbiome in ADHD, ASD and epilepsy

The study demonstrates that fecal microbiota transplantation from individuals with ADHD into mice induces not only shifts in gut microbial composition but also significant alterations in behavior, brain structure, and function (Tengeler et al., 2020). These findings underscore a potential mechanistic link between gut microbiota and early neurodevelopmental processes. Comparative analyses of gut microbiota profiles between children with ADHD and healthy controls revealed distinct dysbiosis patterns, characterized by reduced relative abundances of *Faecalibacterium* and *Veillonellaceae*, alongside increased abundances of *Enterococcus* and *Odoribacter (* Wan et al., 2020). Notably, *Faecalibacterium*, a genus known for its anti-inflammatory properties, is consistently reported at lower levels in inflammatory conditions (Martin et al., 2023), suggesting a possible role in ADHD-related pathophysiology. Further supporting this, Szopinska-Tokov et al. conducted a study involving 42 adolescents and young adults with ADHD, revealing a significant increase in *Ruminococcaceae_UGC_004* and a reduction in β-diversity, which was particularly associated with inattention symptoms (Szopinska-Tokov et al., 2020). Intriguingly, *Ruminococcaceae_UGC_004* shares genetic sequences with microbial species capable of degrading GABA, a key inhibitory neurotransmitter. Additionally, ADHD patients exhibited significantly reduced α diversity (Prehn-Kristensen et al., 2018), which was inversely correlated with hyperactivity severity, further highlighting the interplay between gut microbial diversity and ADHD symptomatology.

Furthermore, extensive research has documented significant alterations in gut microbiota community structure among individuals with ASD, which are closely linked to the high prevalence of gastrointestinal disorders (GIDs) such as diarrhea, constipation, and abdominal pain in this population (Kho and Lal, 2018). A pivotal reseacher by Zhou et al (Dan et al., 2020). revealed a marked reduction in the relative abundance of *Prevotella*, *Prevotella copri*, and *Prevotella stercorea* in the gut microbiota of ASD patients. As a keystone genus in the human gut, *Prevotella* plays a critical role in maintaining intestinal homeostasis and modulating immune responses. Specifically, *Prevotella copri* produces succinic acid, which binds to succinate receptors on dendritic cells, enhancing antigen-specific T cell responses and contributing to host immune defense mechanisms (Dan et al., 2020). Additionally, the fecal microbiota composition in ASD children diverges notably, with Bacteroidetes showing diminished abundance and Firmicutes, *Lactobacillus*, and *Desulfovibrio* demonstrating heightened prevalence (Tomova et al., 2015). Notably, the relative abundance of *Desulfovibrio* and the Bacteroidetes-to-Firmicutes ratio have been shown to correlate with both ASD severity and the intensity of GIDs (Kwak et al., 2023), further underscoring the potential role of gut microbiota in ASD pathophysiology.

Accumulating evidence highlights a significant association between epilepsy and gut microbiota dysbiosis. At the genus level, the relative abundance of *Roseburia* and *Blautia* was reduced (Mousavi et al., 2025). *Roseburia* is one of the major butyrate producers in the gut microbiota, with butyrate showing antiepileptic properties in a model of experimental epilepsy (Lal et al., 2001). Moreover, in a previous study, it was found that children with epilepsy had very low levels of *Blautia* in their intestines (Huang et al., 2019). From another study, it was observed that *Proteobacteria* were higher in epileptic patients than in healthy individuals, and *Campylobacter*, *Delftia*, *Haemophilus*, *Lautropia*, and *Neisseria* among the *Proteobacteria* were significantly elevated in epileptic patients (Safak et al., 2020). Conversely, the relative abundances of Bacteroidetes and Firmicutes, which dominate the healthy gut microbiota, were significantly reduced in epileptic patients (Safak et al., 2020).

#### 4.2.2 Bidirectional regulation of the gut-brain axis

**Figure 2:**
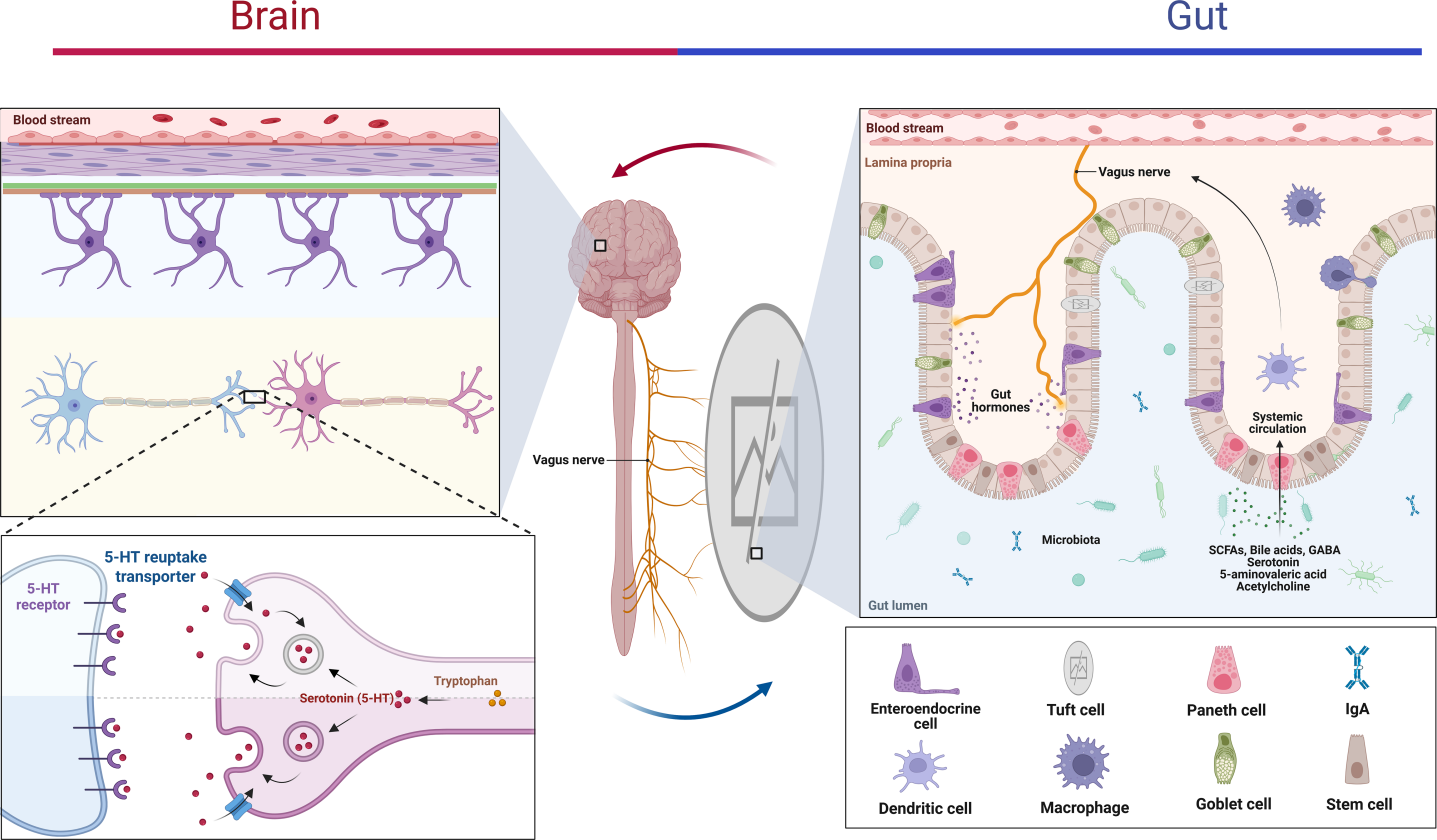
Bidirectional regulation of the gut-brain axis. The gut microbiota interacts bidirectionally with the central nervous system via a neuroendocrine-immune network. This crosstalk occurs either through direct signaling via microbially-derived metabolites or indirectly by modulating gut-derived molecules that activate vagal afferent pathways. Furthermore, the gut microbiota orchestrates 5-HT synthesis within enterochromaffin cells. Notably, 5-HT transport across the BBB requires vagus nerve involvement. Concurrently, the metabolites SCFAs and bile acids and related compounds produced by microorganisms are not only associated with vagal circuits, but also influence ENS activity and gut mechanosensory responses. The vagus nerve serves as a pivotal mediator between endocrine and neuronal signaling pathways.

The gut-brain axis represents an intricate bidirectional communication network connecting the CNS and the GI, integrating neural, endocrine, and immune pathways to regulate neurodevelopment, brain function and cognition (Chen et al., 2021b). Emerging research has elucidated that the CNS communicates with the gut via interconnected neuronal, hormonal, and neuroendocrine pathways, orchestrating key intestinal functions such as motility, permeability, and mucosal immunity (Bicknell et al., 2023). For instance, traumatic brain injury (TBI) induces elevated plasma endotoxin levels and increased intestinal permeability, leading to gut dysbiosis and gastrointestinal dysfunction (Treangen et al., 2018). Similar disruptions in gut microbiota composition have been observed in animal models of cerebral ischemia (Singh et al., 2016) and spinal cord injury (Manrique et al., 2016), underscoring the systemic impact of neurological insults on gut homeostasis. Communication from the gut microbiome to the CNS occurs through two primary modalities: (1) rapid signaling via the vagus nerve, which directly links the enteric nervous system (ENS) to brain regions regulating emotion and cognition; and (2) slower, indirect pathways mediated by the hypothalamic-pituitary-adrenal (HPA) axis, immune responses, and microbial metabolites such as SCFAs, hormones, and tryptophan derivatives (Ullah et al., 2023). Experimental studies demonstrate that gut microbiota depletion or compositional shifts can dysregulate HPA axis activity, altering stress perception and anxiety-related behaviors (Diaz Heijtz et al., 2011). Conversely, glucocorticoids released during HPA axis activation under stress directly modulate ENS function and intestinal inflammation (Schneider et al., 2023), establishing a feedback loop between psychological states and gut physiology. The vagus nerve, a major component of the parasympathetic nervous system, serves as the most direct conduit for gut-brain communication. It regulates intestinal motility, permeability, enzyme secretion, and mucus production while transmitting microbial signals to the CNS (Bonaz et al., 2018). Animal studies reveal that *L. rhamnosus* supplementation modulates emotional behaviors and GABA receptor (GABAAα2, GABAAα1, GABAB1b) mRNA expression via vagus nerve-dependent mechanisms (Bravo et al., 2011), which has a palliative effect on the development and symptoms of ADHD (Partty et al., 2015). Building on this, vagus nerve stimulation has emerged as a therapeutic intervention for NDDs due to its potent anti-inflammatory effects (Wang Q. et al., 2023), highlighting its potential to restore gut-brain axis homeostasis. Immune system involvement in the gut-brain axis is equally critical. The gastrointestinal tract harbors a dense population of immune cells that continuously interact with resident microbiota (Ullah et al., 2025). Dysbiosis-induced immune imbalance can trigger systemic inflammation, exemplified by LPS-mediated activation of immune cells and cytokine release, which promote CNS inflammation by compromising the blood-brain barrier (**Figure 2**) (Bonaz et al., 2018).

##### 4.2.2.1 Probiotics modulate the gut-brain axis to improve ASD

**Figure 3:**
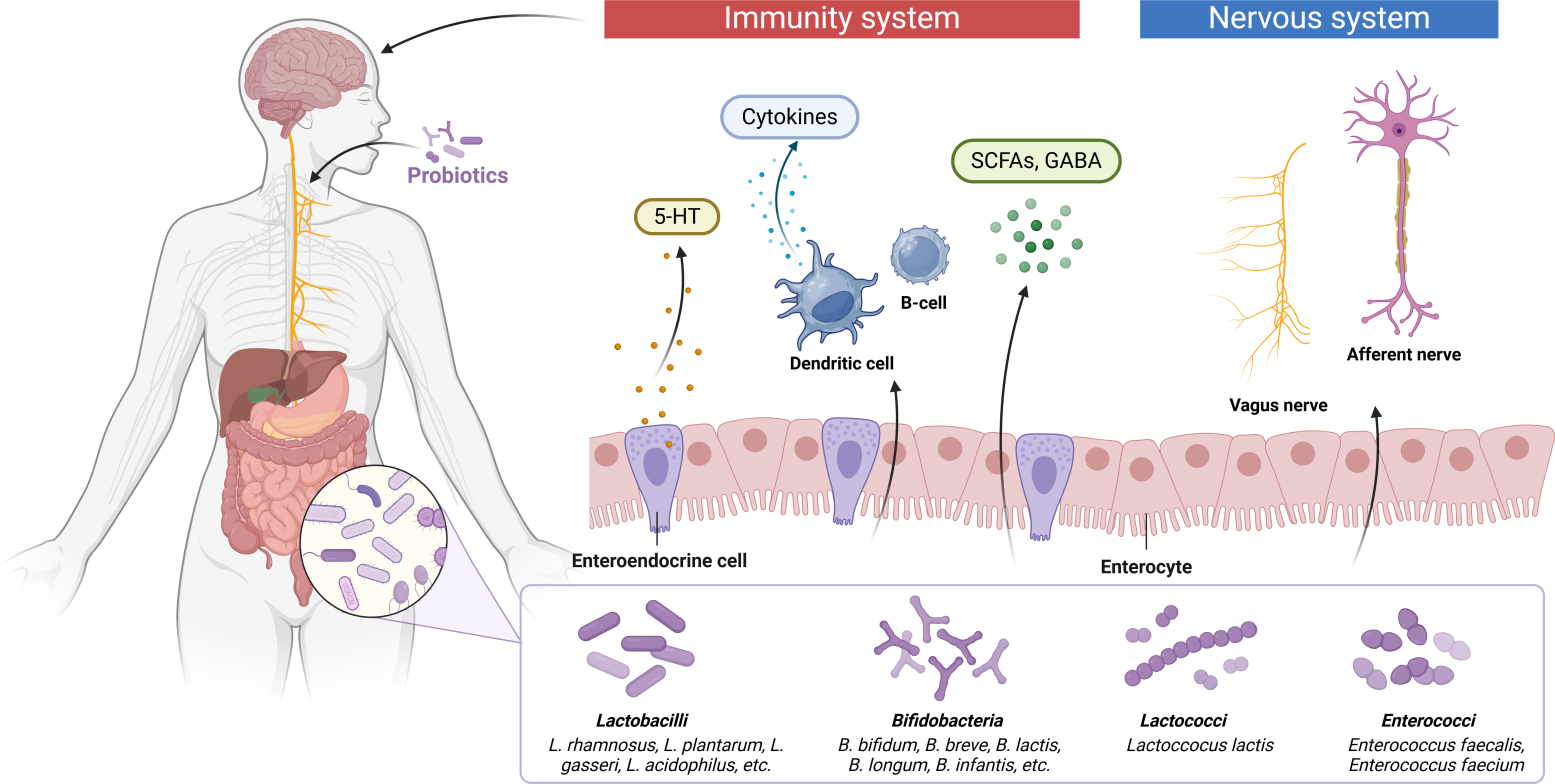
The potential of probiotics and their metabolites to treat ASD through the gut-brain axis. The gut microbiota interacts with the brain through endocrine and neurosecretory pathways, while the brain influences microbial composition through the autonomic nervous system by mechanisms that are also closely linked to the immune and humoral systems. The gut microbiota can ameliorate ASD symptoms either by directly producing neurotransmitter precursors (5-HT, GABA) or by stimulating gut flora metabolites in order to promote synthesis and release of neurotransmitters from enteroendocrine cells.

Accumulating evidence indicates a strong association between ASD pathogenesis and gut-brain axis dysfunction (**Figure 3**). The gut microbiota influences brain function through multiple pathways, including neuroendocrine signaling, neuroimmune modulation, and autonomic nervous system regulation (Goralczyk-Binkowska et al., 2022). Clinically, a significant proportion of ASD patients present with comorbid GIDs, where gut dysbiosis contributes to immune dysregulation. This process involves the release of proinflammatory chemokines and cytokines that can cross the compromised BBB and bind to cerebral endothelial cells, subsequently triggering neuroinflammatory responses (de Theije et al., 2011). Current research supports probiotic modulation of the microbiota-gut-brain axis as a potential therapeutic strategy for ASD (Feng et al., 2023). Shaaban et al. demonstrated that 3-month probiotic supplementation in ASD children led to measurable improvements in both gastrointestinal symptoms and core ASD behaviors, including social interaction and functional severity (Shaaban et al., 2018). Daily administration of *Lactobacillus plantarum* WCFS1 (4.5×10^10^ CFU) significantly enhanced intestinal microbiota composition, particularly increasing *Enterococcus* and *Lactobacillus* populations, while improving gut function (Sivamaruthi et al., 2020). Oral administration of *L. rhamnosus* (1×10^9^ CFU/day for 28 days) modified behavioral responses through vagus nerve-mediated mechanisms (Bravo et al., 2011). Furthermore, ICR mice given *L. plantarum* STIII (5 × 108 CFU/g, 0.8 ml/d) by intragastric tube feeding for two consecutive weeks exhibited improved social impairment, self-effacement, and freezing time. This was accompanied by an increase in the abundance of the beneficial bacterium *Lachnospiraceae* and a decrease in the relative abundance of *Alistipes (* Guo et al., 2022). Prenatal valproic acid-induced ASD models showed reversal of autistic-like behaviors and immune function normalization following *Lactobacillus* supplementation (Feng et al., 2023).

## 5 Role of related signalling pathways in NDDs

### 5.1 NF-κB

NF-κB, a family of transcription factors comprising p65, RelB, c-Rel, NF-κB1, and NF-κB2, serves as a central orchestrator of neuroinflammatory and OS pathways by governing the expression of pro-inflammatory cytokines and OS-associated enzymes (iNOS, COX-2) (Ghosh et al., 2012). Neuroinflammation, a hallmark of NDDs, is primarily driven by microglial activation, which releases a cascade of inflammatory mediators, contributing to neuronal dysfunction and death (Lee et al., 2021). For instance, LPS-induced microglial overactivation triggers the release of NO, TNF-α, IL-1β, and IL-6, leading to neuronal apoptosis and CNS damage (Amor et al., 2010). The NF-κB signaling pathway, activated by cytokine receptors and TLR4, regulates microglial activation and upregulates the expression of iNOS, COX-2, TNF-α, IL-1β and IL-6, thereby amplifying neuroinflammatory responses (Bethea et al., 1998). In a study utilizing the OLINK technique to assess 96 inflammatory proteins in a deeply phenotyped cohort of 126 adults with ADHD, the NF-κB pathway emerged as one of the most significantly altered biological processes, alongside chemokine, IL-17, metabolic dysregulation, and chemokine attraction (Schnorr et al., 2024). Supporting this, Cortese et al. found that elevated levels of IL-6 and TNF-α were positively correlated with hyperactive-impulsive symptoms in children and adolescents with ADHD and comorbid obesity, further implicating NF-κB-mediated inflammation in ADHD pathophysiology (Schnorr et al., 2024). Similarly, in an epileptic mouse model, increased expression of SerpinA3N was shown to exacerbate hippocampal neuroinflammation through NF-κB pathway activation and RYR2 phosphorylation, highlighting the role of NF-κB in epilepsy-related neuroinflammation. Given its central role in neuroinflammation, targeting the NF-κB pathway to downregulate pro-inflammatory cytokine production and enhance the anti-inflammatory functions of microglia represents a promising therapeutic strategy for NDDs. This approach could mitigate neuroinflammatory damage and improve behavioral and cognitive outcomes in conditions such as ADHD, ASD, and epilepsy.

PYC has been reported to act as an inhibitor of the NF-κB. In a study examining the expression of pro-inflammatory factors in LPS-stimulated microglia after PYC treatment, the researchers found a significant reduction in the release of NO, TNF-α, IL-6, and IL-1β, as well as lower levels of intercellular adhesion molecule 1 and perilipin 2, in a dose-dependent manner after PYC treatment (Fan et al., 2015). In an LPS-induced atherosclerosis mouse model, PYC pretreatment significantly inhibited TLR4 expression and NF-κB activation, resulting in a decrease in the number of macrophages accumulating in plaques and a reduction in the levels of pro-inflammatory cytokines (Luo et al., 2015). Jafari F et al. administered PYC to 6-hydroxydopamine-induced Parkinson’s mice at doses of 10, 20, and 30 mg/kg for 7 days and found that PYC treatment increased the expression of Nrf2 anti-inflammatory genes and exhibited neuroprotective effects (Jafari et al., 2022).

### 5.2 MAPK

The mitogen-activated protein kinase (MAPK) signaling pathway, including the c-Jun N-terminal kinases (JNKs), p38, and extracellular signal-regulated kinases (ERK), represents a central signaling cascade regulating cellular apoptosis and synaptic plasticity (Chen et al., 2021a). JNK signaling attenuates OS-induced hippocampal neuronal apoptosis (Zhang et al., 2020). In addition to apoptosis regulation, JNK isoforms regulate neurodevelopmental processes such as cerebral morphogenesis, axodendritic patterning, synaptic plasticity, and memory consolidation (Coffey, 2014). Persistent JNK hyperactivation has been demonstrated to drive epileptogenic by exacerbating post-ictal neuronal apoptosis, neuroinflammation, and hippocampal neurodegeneration (Singh et al., 2021). Notably, JNK3-deficient murine epilepsy models display attenuated seizure activity and neuronal apoptosis, accompanied by reduced hippocampal neurodegeneration, diminished gliosis, and downregulated inflammatory gene expression relative to wild-type controls (Auladell et al., 2017). Similarly, JNK1 knockout models exhibit expanded populations of immature neurons, highlighting distinct isoform-specific functions in neurogenesis (de Lemos et al., 2018). Emerging studies support the functional involvement of p38 MAPK in modulating synaptic plasticity. *In vivo* studies employing p38 heterozygous knockdown mice (p38KI/+) demonstrated that hippocampal p38 MAPK downregulation mitigates angiotensin II-induced cognitive deficits and restores synaptic plasticity (Dai et al., 2016). These findings collectively underscore the therapeutic potential of targeting isoform-specific MAPK signaling for neurodevelopmental and neurodegenerative disorders.

ERK and its phosphorylated forms are critical for neurobehavioral responses and cognitive processes under both physiological and pathological conditions. Evidence indicates that ERK activation drives hippocampal apoptosis, a process linked to the pathogenesis of childhood learning disabilities (Yan et al., 2019). Tight regulation of ERK signaling promotes neural stem cell differentiation into neurons, suppresses apoptotic cascades, and supports synaptic plasticity through the formation of functional neural circuits. PYC has been reported to modulate ERK activity and protect neurons from damage. In a study by Xia et al., primary rat astrocytes were subjected to oxygen-glucose deprivation/reoxygenation (OGD/R) injury and treated with PYC at concentrations of 10, 20, 40, and 60 μg/mL. Cell viability assays and Western blot analysis revealed that PYC attenuated OGD/R-induced cell viability loss and oxidative stress, and inhibited ERK1/2 phosphorylation (Xia et al., 2017). These findings indicate that PYC exerts neuroprotective effects through the ERK1/2 MAPK pathway.

### 5.3 PI3K/AKT/mTOR

**Figure 4:**
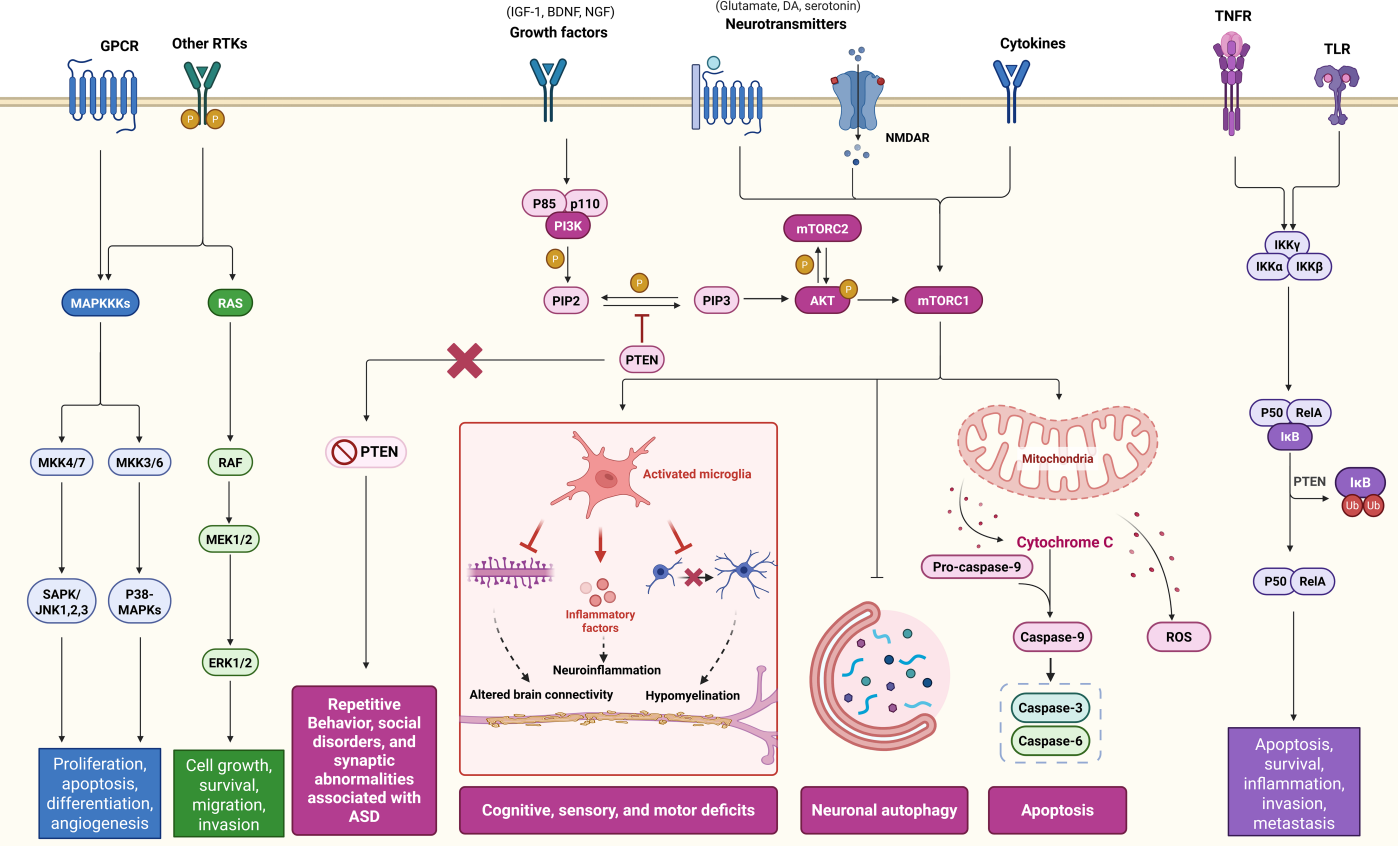
Regulatory mechanisms of NF-κB, MAPK and PI3K/AKT/mTOR. In NDDs, the NF-κB, MAPK and PI3K/AKT/mTOR signaling pathways are central and closely related to apoptosis and proliferation, as well as neuronal survival and inflammation. For PI3K/AKT, PI3K converts PIP2 to PIP3, which recruits Akt kinase and stimulates Akt phosphorylation via mTORC2. mTOR is a key downstream target of the PI3K/Akt pathway and an important autophagy regulator. mTORC1 is a core component downstream of PI3K/Akt, and p-Akt can directly activate mTORC1, making it a key component of PI3K/Akt. mTORC1 is a core component downstream of PI3K/Akt. p-Akt can directly activate mTORC1 to positively regulate translation. p-Akt can also phosphorylate mTORC2, which in turn activates the PI3K/Akt/mTOR pathway to regulate downstream autophagy, and is also involved in neuroinflammation, altered brain connectivity and apoptosis.

The PI3K/AKT/mTOR signaling pathway constitutes a critical regulatory axis in cellular physiology, comprising three core components: phosphoinositide-3 kinase (PI3K), protein kinase B (PKB/AKT), and mammalian target of rapamycin (mTOR) (**Figure 4**). Upon activation by extracellular signals, PI3K catalyzes the phosphorylation of phosphatidylinositol lipids to generate phosphatidylinositol-3,4,5-trisphosphate (PIP3), a secondary messenger essential for AKT membrane recruitment and activation (Jafari et al., 2019). AKT exerts pleiotropic effects on neuronal homeostasis through phosphorylation-dependent modulation of downstream targets, including anti-apoptotic regulators BCL-2 and BCL-XL, thereby orchestrating neuronal survival, proliferation, and differentiation (Hou et al., 2018). This signaling cascade is initiated through receptor tyrosine kinases and cytokine receptors (Jacobs et al., 1993), playing pivotal roles in neurodevelopmental processes such as synaptogenesis, corticogenesis, and cortical circuit assembly (Sharma and Mehan, 2021). Pathological hyperactivation of PI3K/AKT/mTOR signaling induces structural abnormalities including neuronal hypertrophy, axonal guidance defects, and disrupted connectivity patterns across brain regions (Heras-Sandoval et al., 2014). Importantly, mTOR-dependent dysregulation of cortical networks mediating higher cognition has been mechanistically linked to autism spectrum disorder pathogenesis (Sharma and Mehan, 2021). Within the CNS, mTOR integrates diverse physiological functions spanning synaptic plasticity (Sensi et al., 2018), neurogenesis, neuronal migration, memory consolidation, and autophagy regulation (Kassai et al., 2014). Mounting studies have demonstrated that mTOR acts as a pivotal downstream effector molecule of PI3K/AKT in synaptic plasticity. mTOR is capable of targeting and regulating local protein synthesis on prominent functions and structures. It has been reported that mTOR supports synaptic plasticity by phosphorylating eIF4E-bind-ing protein 1 and Ribosomal protein S6 kinase beta-1, thereby promoting ribosomal protein synthesis and mRNA translation (Peterson et al., 2011), which provide the material basis for the formation of post-synaptic dendritic spines and the enhancement of synaptic strength. In addition, Gao et al. found that inhibition of mTOR overactivation modulates autophagy and increases the expression of post-synaptic density markers (PSD-95) and presynaptic vesicle proteins (SYP) to ameliorate anesthesia/surgery-induced memory and cognitive dysfunction by constructing a mice model (Gao et al., 2021). While PTEN functions as a key negative regulator of the PI3K/AKT/mTOR axis (Naderali et al., 2018), its deficiency has been mechanistically linked to core ASD phenotypes. Preclinical evidence demonstrates that PTEN knockout mice recapitulate neurodevelopmental features of ASD, manifesting macrosomia, impaired social preference, cognitive deficits, and heightened seizure susceptibility. Notably, these animal models exhibit synaptic pathophysiology consistent with human ASD cortical circuit abnormalities (Sharma and Mehan, 2021). Emerging regulatory mechanisms further implicate this pathway in neuropsychiatric disorders. A seminal study by Duan et al. identified microRNA-155 as a potent driver of epileptogenic through PI3K/AKT/mTOR hyperactivation, revealing novel cross-talk between non-coding RNAs and canonical signaling pathways in neurological disease pathogenesis (Duan et al., 2018).

Moreover, PYC has been reported to enhance glucose uptake in a dose-dependent manner by promoting the membrane translocation of glucose transporter protein (GLUT4) and increasing the relative abundance of Akt mRNAs through the PI3K pathway (Lee et al., 2010). Meanwhile, procyanidins, the primary components of PYC, have been found to down-regulate the accumulation of reactive oxygen species (ROS) and inhibit the activation of inflammatory factors. Excessive ROS release can lead to aberrant activation of the AKT/mTOR pathway, which in turn triggers inflammation and metabolic disorders (Chen L. et al., 2017). This suggests that procyanidins can indirectly down-regulate the over-activation of the PI3K/AKT/mTOR pathway (Zhao et al., 2022), thereby alleviating neuroinflammation.

## 6 Conclusion and perspectives

The global prevalence of NDDs continues to rise, with their etiology encompassing complex interactions among genetic factors (including inherited and *de novo* mutations), immune dysregulation, and environmental influences. These pathological interactions ultimately lead to neuronal damage, synaptic abnormalities, dysregulation of signaling pathways, immune activation, and disrupted brain functional connectivity. Recently, the bidirectional regulatory potential of the gut-brain axis in NDD treatment has garnered significant attention. Research indicates that gut microbiota and their metabolites can enhance CNS function by modulating chemokine, cytokine, and neurotransmitter (GABA and 5-HT) expression, as well as influencing synaptic plasticity, blood-brain barrier permeability, and neuroinflammatory responses, thereby improving neurodevelopment and behavior. Furthermore, extensive studies have demonstrated a close association between NDDs and GIDs. In this review, we comprehensively examined current literature on NDDs pathological mechanisms and the roles of NF-κB, MAPK, and PI3K/AKT/mTOR signaling pathways in NDDs pathogenesis. We propose that PYC, a pine bark polyphenol extract, can modulate the structure of gut microbial community to increase the expression of SCFAs, GABA and 5-HT targeting the gut-brain axis as well as regulating synaptic protein synthesis and promoting plasticity, thus becoming a powerful natural drug candidate for the treatment of NDDs. Future studies should consider exploring the direct targets of PYC on NF-κB, MAPK and PI3K/AKT/mTOR pathways, such as inhibiting the activity of TLR4 and IKKβ to block the nuclear translocation of NF-κB and reduce the release of inflammatory factors. And PYC regulates mTORC1 complex activity to promote neuronal survival and synaptic plasticity.The combined signaling pathway constructs a visual model of gut flora-metabolite-signaling pathway, as well as linking the gut-brain axis, to clarify the therapeutic strategy of PYC as a drug candidate to mediate the gut-brain axis against NDDs.

## References

[B1] Aguiar C. C. Almeida A. B. Araujo P. V. de Abreu R. N. Chaves E. M. do Vale O. C.. (2012). Oxidative stress and epilepsy: literature review. Oxid. Med. Cell Longev 2012, 795259. doi: 10.1155/2012/795259 22848783 PMC3403512

[B2] Akyuz E. Polat A. K. Eroglu E. Kullu I. Angelopoulou E. Paudel Y. N.. (2021). Revisiting the role of neurotransmitters in epilepsy: An updated review. Life Sci. 265, 118826. doi: 10.1016/j.lfs.2020.118826 33259863

[B3] Alderton G. Scanlon S. T. (2021). Inflammation. Science 374, 1068–1069. doi: 10.1126/science.abn1721 34822288

[B4] Amor S. Puentes F. Baker D. van der Valk P.. (2010). Inflammation in neurodegenerative diseases. Immunology 129, 154–169. doi: 10.1111/j.1365-2567.2009.03225.x 20561356 PMC2814458

[B5] Arshad M. N. Pinto A. van Praag H. Naegele J. R.. (2023). Altered connectomes of adult-born granule cells following engraftment of GABAergic progenitors in the mouse hippocampus. Prog. Neurobiol. 226, 102450. doi: 10.1016/j.pneurobio.2023.102450 37061022 PMC11351537

[B6] Auladell C. de Lemos L. Verdaguer E. Ettcheto M. Busquets O. Lazarowski A.. (2017). Role of JNK isoforms in the kainic acid experimental model of epilepsy and neurodegeneration. Front. Biosci (Landmark Ed) 22, 795–814. doi: 10.2741/4517 27814647

[B7] Ayano G. Demelash S. Gizachew Y. Tsegay L. Alati R.. (2023). The global prevalence of attention deficit hyperactivity disorder in children and adolescents: An umbrella review of meta-analyses. J. Affect. Disord. 339, 860–866. doi: 10.1016/j.jad.2023.07.071 37495084

[B8] Bayer J. Hogger P. (2024). Review of the pharmacokinetics of French maritime pine bark extract (Pycnogenol((R))) in humans. Front. Nutr. 11, 1389422. doi: 10.3389/fnut.2024.1389422 38757126 PMC11096517

[B9] Belviranli M. Okudan N. (2015). “Well-known antioxidants and newcomers in sport nutrition: coenzyme Q10, quercetin, resveratrol, pterostilbene, pycnogenol and astaxanthin,” in Antioxidants in sport nutrition. Ed. Lamprecht M.(Boca Raton (FL). 26065085

[B10] Bethea J. R. Castro M. Keane R. W. Lee T. T. Dietrich W. D. Yezierski R. P.. (1998). Traumatic spinal cord injury induces nuclear factor-kappaB activation. J. Neurosci. 18, 3251–3260. doi: 10.1523/JNEUROSCI.18-09-03251.1998 9547234 PMC6792666

[B11] Bicknell B. Liebert A. Borody T. Herkes G. McLachlan C. Kiat H.. (2023). Neurodegenerative and neurodevelopmental diseases and the gut-brain axis: the potential of therapeutic targeting of the microbiome. Int. J. Mol. Sci. 24. doi: 10.3390/ijms24119577 PMC10253993 37298527

[B12] Bidwell L. C. McClernon F. J. Kollins S. H. (2011). Cognitive enhancers for the treatment of ADHD. Pharmacol. Biochem. Behav. 99, 262–274. doi: 10.1016/j.pbb.2011.05.002 21596055 PMC3353150

[B13] Bonaz B. Bazin T. Pellissier S. (2018). The vagus nerve at the interface of the microbiota-gut-brain axis. Front. Neurosci. 12, 49. doi: 10.3389/fnins.2018.00049 29467611 PMC5808284

[B14] Bonisch H. Bruss M. (2006). The norepinephrine transporter in physiology and disease. Handb. Exp. Pharmacol. 175, 485–524. doi: 10.1007/3-540-29784-7_20 16722247

[B15] Brackenbury W. J. Isom L. L. (2011). Na channel beta subunits: overachievers of the ion channel family. Front. Pharmacol. 2, 53. doi: 10.3389/fphar.2011.00053 22007171 PMC3181431

[B16] Bravo J. A.. (2011). Ingestion of Lactobacillus strain regulates emotional behavior and central GABA receptor expression in a mouse via the vagus nerve. Proc. Natl. Acad. Sci. U S A 108, 16050–16055. doi: 10.1073/pnas.1102999108 21876150 PMC3179073

[B17] Brescia P. Rescigno M. (2021). The gut vascular barrier: a new player in the gut-liver-brain axis. Trends Mol. Med. 27, 844–855. doi: 10.1016/j.molmed.2021.06.007 34229973

[B18] Bulut M. Selek S. Gergerlioglu H. S. Savas H. A. Yilmaz H. R. Yuce M.. (2007). Malondialdehyde levels in adult attention-deficit hyperactivity disorder. J. Psychiatry Neurosci. 32, 435–438. 18043768 PMC2077350

[B19] Buske-Kirschbaum A. Schmitt J. Plessow F. Romanos M. Weidinger S. Roessner V.. (2013). Psychoendocrine and psychoneuroimmunological mechanisms in the comorbidity of atopic eczema and attention deficit/hyperactivity disorder. Psychoneuroendocrinology 38, 12–23. doi: 10.1016/j.psyneuen.2012.09.017 23141851

[B20] Carbonero F. Benefiel A. C. Alizadeh-Ghamsari A. H. Gaskins H. R.. (2012). Microbial pathways in colonic sulfur metabolism and links with health and disease. Front. Physiol. 3, 448. doi: 10.3389/fphys.2012.00448 23226130 PMC3508456

[B21] Castellanos F. X. Lee P. P. Sharp W. Jeffries N. O. Greenstein D. K. Clasen L. S.. (2002). Developmental trajectories of brain volume abnormalities in children and adolescents with attention-deficit/hyperactivity disorder. JAMA 288, 1740–1748. doi: 10.1001/jama.288.14.1740 12365958

[B22] Cenit M. C. Nuevo I. C. Codoner-Franch P. Dinan T. G. Sanz Y.. (2017). Gut microbiota and attention deficit hyperactivity disorder: new perspectives for a challenging condition. Eur. Child Adolesc. Psychiatry 26, 1081–1092. doi: 10.1007/s00787-017-0969-z 28289903

[B23] Ceylan M. Sener S. Bayraktar A. C. Kavutcu M.. (2010). Oxidative imbalance in child and adolescent patients with attention-deficit/hyperactivity disorder. Prog. Neuropsychopharmacol. Biol. Psychiatry 34, 1491–1494. doi: 10.1016/j.pnpbp.2010.08.010 20732373

[B24] Chakraborty S. Parayil R. Mishra S. Nongthomba U. Clement J. P.. (2022). Epilepsy characteristics in neurodevelopmental disorders: research from patient cohorts and animal models focusing on autism spectrum disorder. Int. J. Mol. Sci. 23. doi: 10.3390/ijms231810807 PMC9501968 36142719

[B25] Chanda S. Aoto J. Lee S. J. Wernig M. Sudhof T. C.. (2016). Pathogenic mechanism of an autism-associated neuroligin mutation involves altered AMPA-receptor trafficking. Mol. Psychiatry 21, 169–177. doi: 10.1038/mp.2015.20 25778475 PMC4573762

[B26] Chen R. Davis L. K. Guter S. Wei Q. Jacob S. Potter M. H.. (2017). Leveraging blood serotonin as an endophenotype to identify *de novo* and rare variants involved in autism. Mol. Autism 8, 14. doi: 10.1186/s13229-017-0130-3 28344757 PMC5361831

[B27] Chen L. Liu P. Feng X. Ma C.. (2017). Salidroside suppressing LPS-induced myocardial injury by inhibiting ROS-mediated PI3K/Akt/mTOR pathway *in vitro* and *in vivo* . J. Cell Mol. Med. 21, 3178–3189. doi: 10.1111/jcmm.2017.21.issue-12 28905500 PMC5706507

[B28] Chen Y. Li L. Zhang J. Cui H. Wang J. Wang C.. (2021a). Dexmedetomidine Alleviates Lipopolysaccharide-Induced Hippocampal Neuronal Apoptosis via Inhibiting the p38 MAPK/c-Myc/CLIC4 Signaling Pathway in Rats. Mol. Neurobiol. 58, 5533–5547. doi: 10.1007/s12035-021-02512-9 34363182

[B29] Chen G. T. Geschwind D. H. (2022). Challenges and opportunities for precision medicine in neurodevelopmental disorders. Adv. Drug Delivery Rev. 191, 114564. doi: 10.1016/j.addr.2022.114564 PMC10409256 36183905

[B30] Chen Y. Xu J. Chen Y. (2021b). Regulation of neurotransmitters by the gut microbiota and effects on cognition in neurological disorders. Nutrients 13. doi: 10.3390/nu13062099 PMC8234057 34205336

[B31] Cheng Y. S. Chen Z. T. Liao T. Y. Lin C. Shen H. C. Wang Y. H.. (2017). An intranasally delivered peptide drug ameliorates cognitive decline in Alzheimer transgenic mice. EMBO Mol. Med. 9, 703–715. doi: 10.15252/emmm.201606666 28356312 PMC5412883

[B32] Cheng L. H. Liu Y. W. Wu C. C. Wang S. Tsai Y. C.. (2019). Psychobiotics in mental health, neurodegenerative and neurodevelopmental disorders. J. Food Drug Anal. 27, 632–648. doi: 10.1016/j.jfda.2019.01.002 31324280 PMC9307042

[B33] Christodoulou G. N. Margariti M. Christodoulou N. (2018). Delusional misidentifications in a procrustean bed. Psychiatriki 29, 15–18. doi: 10.22365/jpsych.2018.291.15 29754115

[B34] Coffey E. T. (2014). Nuclear and cytosolic JNK signalling in neurons. Nat. Rev. Neurosci. 15, 285–299. doi: 10.1038/nrn3729 24739785

[B35] Corona J. C. (2020). Role of oxidative stress and neuroinflammation in attention-deficit/hyperactivity disorder. Antioxidants (Basel) 9. doi: 10.3390/antiox9111039 PMC7690797 33114154

[B36] Cortese S. Coghill D. (2018). Twenty years of research on attention-deficit/hyperactivity disorder (ADHD): looking back, looking forward. Evid Based Ment Health 21, 173–176. doi: 10.1136/ebmental-2018-300050 30301823 PMC10270437

[B37] Cossins E. Lee R. Packer L. (1998). ESR studies of vitamin C regeneration, order of reactivity of natural source phytochemical preparations. Biochem. Mol. Biol. Int. 45, 583–597. doi: 10.1080/15216549800202982 9679660

[B38] Costales J. L. Kolevzon A. (2015). Phelan-mcDermid syndrome and SHANK3: implications for treatment. Neurotherapeutics 12, 620–630. doi: 10.1007/s13311-015-0352-z 25894671 PMC4489957

[B39] Curatolo P. Paloscia C. D'Agati E. Moavero R. Pasini A.. (2009). The neurobiology of attention deficit/hyperactivity disorder. Eur. J. Paediatr. Neurol. 13, 299–304. doi: 10.1016/j.ejpn.2008.06.003 18644740

[B40] D’Andrea G. (2010). Pycnogenol: a blend of procyanidins with multifaceted therapeutic applications? Fitoterapia 81, 724–736. doi: 10.1016/j.fitote.2010.06.011 20598812

[B41] Dai H. L. Hu W. Y. Jiang L. H. Li L. Gaung X. F. Xiao Z. C.. (2016). p38 MAPK inhibition improves synaptic plasticity and memory in angiotensin II-dependent hypertensive mice. Sci. Rep. 6, 27600. doi: 10.1038/srep27600 27283322 PMC4901328

[B42] Dan Z. Mao X. Liu Q. Guo M. Zhuang Y. Liu Z.. (2020). Altered gut microbial profile is associated with abnormal metabolism activity of Autism Spectrum Disorder. Gut Microbes 11, 1246–1267. doi: 10.1080/19490976.2020.1747329 32312186 PMC7524265

[B43] da Silveira V. T. da Silveira V. T. Medeiros D. C. Ropke J. Guidine P. A. Rezende G. H. Moraes M. F.. (2017). Effects of early or late prenatal immune activation in mice on behavioral and neuroanatomical abnormalities relevant to schizophrenia in the adulthood. Int. J. Dev. Neurosci. 58, 1–8. doi: 10.1016/j.ijdevneu.2017.01.009 28122258

[B44] Davias A. Davias A. Verghese M. Bridgman S. L. Tun H. M. Field C. J. Hicks M.. (2025). Gut microbiota metabolites, secretory immunoglobulin A and Bayley-III cognitive scores in children from the CHILD Cohort Study. Brain Behav. Immun. Health 44, 100946. doi: 10.1016/j.bbih.2025.100946 39911944 PMC11795817

[B45] Daws L. C. Gould G. G. (2011). Ontogeny and regulation of the serotonin transporter: providing insights into human disorders. Pharmacol. Ther. 131, 61–79. doi: 10.1016/j.pharmthera.2011.03.013 21447358 PMC3131109

[B46] de Lemos L. Junyent F. Camins A. Castro-Torres R. D. Folch J. Olloquequi J.. (2018). Neuroprotective effects of the absence of JNK1 or JNK3 isoforms on kainic acid-induced temporal lobe epilepsy-like symptoms. Mol. Neurobiol. 55, 4437–4452. doi: 10.1007/s12035-017-0669-1 28664455

[B47] Deng F. Yang D. Qing L. Chen Y. Zou J. Jia M.. (2025). Exploring the interaction between the gut microbiota and cyclic adenosine monophosphate-protein kinase A signaling pathway: a potential therapeutic approach for neurodegenerative diseases. Neural Regener. Res. 20, 3095–3112. doi: 10.4103/NRR.NRR-D-24-00607 PMC11881707 39589173

[B48] de Theije C. G. Wu J. da Silva S. L. Kamphuis P. J. Garssen J. Korte S. M.. (2011). Pathways underlying the gut-to-brain connection in autism spectrum disorders as future targets for disease management. Eur. J. Pharmacol. 668 Suppl 1, S70–S80. doi: 10.1016/j.ejphar.2011.07.013 21810417

[B49] Diaz Heijtz R. Wang S. Anuar F. Qian Y. Bjorkholm B. Samuelsson A.. (2011). Normal gut microbiota modulates brain development and behavior. Proc. Natl. Acad. Sci. U S A 108, 3047–3052. doi: 10.1073/pnas.1010529108 21282636 PMC3041077

[B50] Dicks L. M. T. (2022). Gut bacteria and neurotransmitters. Microorganisms 10. doi: 10.3390/microorganisms10091838 PMC9504309 36144440

[B51] Doifode T. Giridharan V. V. Generoso J. S. Bhatti G. Collodel A. Schulz P. E.. (2021). The impact of the microbiota-gut-brain axis on Alzheimer’s disease pathophysiology. Pharmacol. Res. 164, 105314. doi: 10.1016/j.phrs.2020.105314 33246175

[B52] Duan W. Chen Y. Wang X. R. (2018). MicroRNA−155 contributes to the occurrence of epilepsy through the PI3K/Akt/mTOR signaling pathway. Int. J. Mol. Med. 42, 1577–1584. doi: 10.3892/ijmm.2018.3711 29901097

[B53] Durak O. Gao F. Kaeser-Woo Y. J. Rueda R. Martorell A. J. Nott A.. (2016). Chd8 mediates cortical neurogenesis via transcriptional regulation of cell cycle and Wnt signaling. Nat. Neurosci. 19, 1477–1488. doi: 10.1038/nn.4400 27694995 PMC5386887

[B54] Dvorakova M. Sivonova M. Trebaticka J. Skodacek I. Waczulikova I. Muchova J.. (2006). The effect of polyphenolic extract from pine bark, Pycnogenol on the level of glutathione in children suffering from attention deficit hyperactivity disorder (ADHD). Redox Rep. 11, 163–172. doi: 10.1179/135100006X116664 16984739

[B55] Dvorakova M. Jezova D. Blazicek P. Trebaticka J. Skodacek I. Suba J.. (2007). Urinary catecholamines in children with attention deficit hyperactivity disorder (ADHD): modulation by a polyphenolic extract from pine bark (pycnogenol). Nutr. Neurosci. 10, 151–157. doi: 10.1080/09513590701565443 18019397

[B56] Ellegood J. Lerch J. P. Henkelman R. M. (2011). Brain abnormalities in a Neuroligin3 R451C knockin mouse model associated with autism. Autism Res. 4, 368–376. doi: 10.1002/aur.v4.5 21882360

[B57] Ellwanger J. H. Franke S. I. Bordin D. L. Pra D. Henriques J. A.. (2016). Biological functions of selenium and its potential influence on Parkinson’s disease. Acad. Bras Cienc 88, 1655–1674. doi: 10.1590/0001-3765201620150595 27556332

[B58] Emanuele E. Orsi P. Boso M. Broglia D. Brondino N. Barale F.. (2010). Low-grade endotoxemia in patients with severe autism. Neurosci. Lett. 471, 162–165. doi: 10.1016/j.neulet.2010.01.033 20097267

[B59] Faltraco F. Palm D. Uzoni A. Borchert L. Simon F. Tucha O.. (2021). Dopamine adjusts the circadian gene expression of Per2 and Per3 in human dermal fibroblasts from ADHD patients. J. Neural Transm (Vienna) 128, 1135–1145. doi: 10.1007/s00702-021-02374-4 34275001 PMC8295132

[B60] Fan B. Dun S. H. Gu J. Q. Guo Y. Ikuyama S.. (2015). Pycnogenol attenuates the release of proinflammatory cytokines and expression of perilipin 2 in lipopolysaccharide-stimulated microglia in part via inhibition of NF-kappaB and AP-1 activation. PloS One 10, e0137837. doi: 10.1371/journal.pone.0137837 26367267 PMC4569068

[B61] Faraone S. V. Asherson P. Banaschewski T. Biederman J. Buitelaar J. K. Ramos-Quiroga J. A.. (2015). Attention-deficit/hyperactivity disorder. Nat. Rev. Dis. Primers 1, 15020. doi: 10.1038/nrdp.2015.20 27189265

[B62] Faraone S. V. Larsson H. (2019). Genetics of attention deficit hyperactivity disorder. Mol. Psychiatry 24, 562–575. doi: 10.1038/s41380-018-0070-0 29892054 PMC6477889

[B63] Feng P. Zhao S. Zhang Y. Li E.. (2023). A review of probiotics in the treatment of autism spectrum disorders: Perspectives from the gut-brain axis. Front. Microbiol 14, 1123462. doi: 10.3389/fmicb.2023.1123462 37007501 PMC10060862

[B64] Follwaczny P. Schieweck R. Riedemann T. Demleitner A. Straub T. Klemm A. H.. (2017). Pumilio2-deficient mice show a predisposition for epilepsy. Dis. Model Mech. 10, 1333–1342. doi: 10.1242/dmm.029678 29046322 PMC5719250

[B65] Frances L. Quintero J. Fernandez A. Ruiz A. Caules J. Fillon G.. (2022). Current state of knowledge on the prevalence of neurodevelopmental disorders in childhood according to the DSM-5: a systematic review in accordance with the PRISMA criteria. Child Adolesc. Psychiatry Ment Health 16, 27. doi: 10.1186/s13034-022-00462-1 35361232 PMC8973738

[B66] Gao S. Zhang S. Zhou H. Tao X. Ni Y. Pei D.. (2021). Role of mTOR-regulated autophagy in synaptic plasticity related proteins downregulation and the reference memory deficits induced by anesthesia/surgery in aged mice. Front. Aging Neurosci. 13, 628541. doi: 10.3389/fnagi.2021.628541 33935683 PMC8085306

[B67] Geier D. A. Kern J. K. Garver C. R. Adams J. B. Audhya T. Geier M. R.. (2009). A prospective study of transsulfuration biomarkers in autistic disorders. Neurochem. Res. 34, 386–393. doi: 10.1007/s11064-008-9782-x 18612812

[B68] Ghosh G. Wang V. Y. Huang D. B. Fusco A.. (2012). NF-kappaB regulation: lessons from structures. Immunol. Rev. 246, 36–58. doi: 10.1111/j.1600-065X.2012.01097.x 22435546 PMC4543363

[B69] Gilbert J. Man H. Y. (2017). Fundamental elements in autism: from neurogenesis and neurite growth to synaptic plasticity. Front. Cell Neurosci. 11, 359. doi: 10.3389/fncel.2017.00359 29209173 PMC5701944

[B70] Goel R. Saxena P. (2019). Pycnogenol protects against pentylenetetrazole-induced oxidative stress and seizures in mice. Curr. Clin. Pharmacol. 14, 68–75. doi: 10.2174/1574884714666181122110317 30465512

[B71] Goralczyk-Binkowska A. Szmajda-Krygier D. Kozlowska E. (2022). The microbiota-gut-brain axis in psychiatric disorders. Int. J. Mol. Sci. 23. doi: 10.3390/ijms231911245 PMC9570195 36232548

[B72] Grayton H. M. Missler M. Collier D. A. Fernandes C.. (2013). Altered social behaviours in neurexin 1alpha knockout mice resemble core symptoms in neurodevelopmental disorders. PloS One 8, e67114. doi: 10.1371/journal.pone.0067114 23840597 PMC3696036

[B73] Grimm T. Skrabala R. Chovanova Z. Muchova J. Sumegova K. Liptakova A.. (2006). Single and multiple dose pharmacokinetics of maritime pine bark extract (pycnogenol) after oral administration to healthy volunteers. BMC Clin. Pharmacol. 6, 4. doi: 10.1186/1472-6904-6-4 16887024 PMC1559639

[B74] Guerrini R. Conti V. Mantegazza M. Balestrini S. Galanopoulou A. S. Benfenati F.. (2023). Developmental and epileptic encephalopathies: from genetic heterogeneity to phenotypic continuum. Physiol. Rev. 103, 433–513. doi: 10.1152/physrev.00063.2021 35951482 PMC9576177

[B75] Gul M. K. Sener E. F. Onal M. G. Demirci E.. (2022). Role of the norepinephrine transporter polymorphisms in atomoxetine treatment: From response to side effects in children with ADHD. J. Psychopharmacol. 36, 715–722. doi: 10.1177/02698811211015245 33944622

[B76] Guo M. Li R. Wang Y. Ma S. Zhang Y. Li S.. (2022). Lactobacillus plantarum ST-III modulates abnormal behavior and gut microbiota in a mouse model of autism spectrum disorder. Physiol. Behav. 257, 113965. doi: 10.1016/j.physbeh.2022.113965 36126693

[B77] Gustafsson H. C. Sullivan E. L. Battison E. A. J. Holton K. F. Graham A. M. Karalunas S. L.. (2020). Evaluation of maternal inflammation as a marker of future offspring ADHD symptoms: A prospective investigation. Brain Behav. Immun. 89, 350–356. doi: 10.1016/j.bbi.2020.07.019 32707260 PMC7703804

[B78] Gvozdjakova A. Kucharska J. Ostatnikova D. Babinska K. Nakladal D. Crane F. L.. (2014). Ubiquinol improves symptoms in children with autism. Oxid. Med. Cell Longev 2014, 798957. doi: 10.1155/2014/798957 24707344 PMC3953391

[B79] Heras-Sandoval D. Perez-Rojas J. M. Hernandez-Damian J. Pedraza-Chaverri J.. (2014). The role of PI3K/AKT/mTOR pathway in the modulation of autophagy and the clearance of protein aggregates in neurodegeneration. Cell Signal 26, 2694–2701. doi: 10.1016/j.cellsig.2014.08.019 25173700

[B80] Hirota T. King B. H. (2023). Autism spectrum disorder: A review. JAMA 329, 157–168. doi: 10.1001/jama.2022.23661 36625807

[B81] Hoogland I. C. Houbolt C. van Westerloo D. J. van Gool W. A. van de Beek D.. (2015). Systemic inflammation and microglial activation: systematic review of animal experiments. J. Neuroinflammation 12, 114. doi: 10.1186/s12974-015-0332-6 26048578 PMC4470063

[B82] Hou Y. Wang K. Wan W. Cheng Y. Pu X. Ye X.. (2018). Resveratrol provides neuroprotection by regulating the JAK2/STAT3/PI3K/AKT/mTOR pathway after stroke in rats. Genes Dis. 5, 245–255. doi: 10.1016/j.gendis.2018.06.001 30320189 PMC6176158

[B83] Hsiao E. Y. McBride S. W. Hsien S. Sharon G. Hyde E. R. McCue T.. (2013). Microbiota modulate behavioral and physiological abnormalities associated with neurodevelopmental disorders. Cell 155, 1451–1463. doi: 10.1016/j.cell.2013.11.024 24315484 PMC3897394

[B84] Hu T. Dong Y. He C. Zhao M. He Q.. (2020). The gut microbiota and oxidative stress in autism spectrum disorders (ASD). Oxid. Med. Cell Longev 2020, 8396708. doi: 10.1155/2020/8396708 33062148 PMC7547345

[B85] Huang C. Li Y. Feng X. Li D. Li X. Ouyang Q.. (2019). Distinct gut microbiota composition and functional category in children with cerebral palsy and epilepsy. Front. Pediatr. 7, 394. doi: 10.3389/fped.2019.00394 31646147 PMC6779726

[B86] Hughes H. K. Moreno R. J. Ashwood P. (2023). Innate immune dysfunction and neuroinflammation in autism spectrum disorder (ASD). Brain Behav. Immun. 108, 245–254. doi: 10.1016/j.bbi.2022.12.001 36494048

[B87] Hughes H. K. Rose D. Ashwood P. (2018). The gut microbiota and dysbiosis in autism spectrum disorders. Curr. Neurol. Neurosci. Rep. 18, 81. doi: 10.1007/s11910-018-0887-6 30251184 PMC6855251

[B88] Human Microbiome Project C. (2012). Structure, function and diversity of the healthy human microbiome. Nature 486, 207–214. doi: 10.1038/nature11234 22699609 PMC3564958

[B89] Jacobs C. A. Beckmann M. P. Mohler K. Maliszewski C. R. Fanslow W. C. Lynch D. H.. (1993). Pharmacokinetic parameters and biodistribution of soluble cytokine receptors. Int. Rev. Exp. Pathol. 34 Pt B, 123–135. doi: 10.1016/B978-0-12-364935-5.50013-4 8384610

[B90] Jafari M. Ghadami E. Dadkhah T. Akhavan-Niaki H.. (2019). PI3k/AKT signaling pathway: Erythropoiesis and beyond. J. Cell Physiol. 234, 2373–2385. doi: 10.1002/jcp.v234.3 30192008

[B91] Jafari F. Goudarzvand M. Hajikhani R. Qorbani M. Solati J.. (2022). Pycnogenol ameliorates motor function and gene expressions of NF-kB and Nrf2 in a 6-hydroxydopamine-induced experimental model of Parkinson’s disease in male NMRI mice. Naunyn Schmiedebergs Arch. Pharmacol. 395, 305–313. doi: 10.1007/s00210-022-02201-x 35024909

[B92] Jakobi B. Vlaming P. Mulder D. Ribases M. Richarte V. Ramos-Quiroga J. A.. (2024). The gut-microbiome in adult Attention-deficit/hyperactivity disorder - A Meta-analysis. Eur. Neuropsychopharmacol. 88, 21–29. doi: 10.1016/j.euroneuro.2024.07.004 39121711

[B93] Kang D. W. Park J. G. Ilhan Z. E. Wallstrom G. Labaer J. Adams J. B.. (2013). Reduced incidence of Prevotella and other fermenters in intestinal microflora of autistic children. PloS One 8, e68322. doi: 10.1371/journal.pone.0068322 23844187 PMC3700858

[B94] Kassai H. Sugaya Y. Noda S. Nakao K. Maeda T. Kano M.. (2014). Selective activation of mTORC1 signaling recapitulates microcephaly, tuberous sclerosis, and neurodegenerative diseases. Cell Rep. 7, 1626–1639. doi: 10.1016/j.celrep.2014.04.048 24857653

[B95] Kebir O. Joober R. (2011). Neuropsychological endophenotypes in attention-deficit/hyperactivity disorder: a review of genetic association studies. Eur. Arch. Psychiatry Clin. Neurosci. 261, 583–594. doi: 10.1007/s00406-011-0207-5 21409419

[B96] Kho Z. Y. Lal S. K. (2018). The human gut microbiome - A potential controller of wellness and disease. Front. Microbiol 9, 1835. doi: 10.3389/fmicb.2018.01835 30154767 PMC6102370

[B97] Kim Y. S. Choi J. Yoon B. E. (2020). Neuron-glia interactions in neurodevelopmental disorders. Cells 9. doi: 10.3390/cells9102176 PMC7601502 32992620

[B98] Kim B. Lee T. K. Park C. W. Kim D. W. Ahn J. H. Sim H.. (2020). Pycnogenol((R)) supplementation attenuates memory deficits and protects hippocampal CA1 pyramidal neurons via antioxidative role in a gerbil model of transient forebrain ischemia. Nutrients 12. doi: 10.3390/nu12082477 PMC7468866 32824513

[B99] Koirala S. Grimsrud G. Mooney M. A. Larsen B. Feczko E. Elison J. T.. (2024). Neurobiology of attention-deficit hyperactivity disorder: historical challenges and emerging frontiers. Nat. Rev. Neurosci. 25, 759–775. doi: 10.1038/s41583-024-00869-z 39448818 PMC13404203

[B100] Kwak M. J. Kim S. H. Kim H. H. Tanpure R. Kim J. I. Jeon B. H.. (2023). Psychobiotics and fecal microbial transplantation for autism and attention-deficit/hyperactivity disorder: microbiome modulation and therapeutic mechanisms. Front. Cell Infect. Microbiol 13, 1238005. doi: 10.3389/fcimb.2023.1238005 37554355 PMC10405178

[B101] Kwan V. Unda B. K. Singh K. K. (2016). Wnt signaling networks in autism spectrum disorder and intellectual disability. J. Neurodev Disord. 8, 45. doi: 10.1186/s11689-016-9176-3 27980692 PMC5137220

[B102] Lal S. Kirkup A. J. Brunsden A. M. Thompson D. G. Grundy D.. (2001). Vagal afferent responses to fatty acids of different chain length in the rat. Am. J. Physiol. Gastrointest Liver Physiol. 281, G907–G915. doi: 10.1152/ajpgi.2001.281.4.G907 11557510

[B103] Langworth-Green C. Patel S. Jaunmuktane Z. Jabbari E. Morris H. Thom M.. (2023). Chronic effects of inflammation on tauopathies. Lancet Neurol. 22, 430–442. doi: 10.1016/S1474-4422(23)00038-8 37059510

[B104] Laria J. C. Delgado-Gomez D. Penuelas-Calvo I. Baca-Garcia E. Lillo R. E.. (2021). Accurate prediction of children’s ADHD severity using family burden information: A neural lasso approach. Front. Comput. Neurosci. 15, 674028. doi: 10.3389/fncom.2021.674028 34234664 PMC8255467

[B105] Lee H. H. Kim K. J. Lee O. H. Lee B. Y.. (2010). Effect of pycnogenol on glucose transport in mature 3T3-L1 adipocytes. Phytother. Res. 24, 1242–1249. doi: 10.1002/ptr.v24:8 20658573

[B106] Lee S. Ju I. G. Choi Y. Park S. Oh M. S.. (2021). Trichosanthis semen suppresses lipopolysaccharide-induced neuroinflammation by regulating the NF-kappaB signaling pathway and HO-1 expression in microglia. Toxins (Basel) 13. doi: 10.3390/toxins13120898 PMC8704237 34941735

[B107] Lei B. Mace B. Dawson H. N. Warner D. S. Laskowitz D. T. James M. L.. (2014). Anti-inflammatory effects of progesterone in lipopolysaccharide-stimulated BV-2 microglia. PloS One 9, e103969. doi: 10.1371/journal.pone.0103969 25080336 PMC4117574

[B108] Li Y. Yin A. Sun X. Zhang M. Zhang J. Wang P.. (2017). Deficiency of tumor suppressor NDRG2 leads to attention deficit and hyperactive behavior. J. Clin. Invest. 127, 4270–4284. doi: 10.1172/JCI94455 29058689 PMC5707150

[B109] Li Z. Zhu Y. X. Gu L. J. Cheng Y.. (2021). Understanding autism spectrum disorders with animal models: applications, insights, and perspectives. Zool Res. 42, 800–824. doi: 10.24272/j.issn.2095-8137.2021.251 34755500 PMC8645879

[B110] Li W. Pozzo-Miller L. (2020). Dysfunction of the corticostriatal pathway in autism spectrum disorders. J. Neurosci. Res. 98, 2130–2147. doi: 10.1002/jnr.v98.11 31758607 PMC7242149

[B111] Liang L. P. Waldbaum S. Rowley S. Huang T. T. Day B. J. Patel M.. (2012). Mitochondrial oxidative stress and epilepsy in SOD2 deficient mice: attenuation by a lipophilic metalloporphyrin. Neurobiol. Dis. 45, 1068–1076. doi: 10.1016/j.nbd.2011.12.025 22200564 PMC3418969

[B112] Liu H. Shi J. Liu F. Zhang L.. (2024). Integrating network pharmacology and experimental verification to reveal the anti-inflammatory ingredients and molecular mechanism of pycnogenol. Front. Pharmacol. 15, 1408304. doi: 10.3389/fphar.2024.1408304 38989153 PMC11233470

[B113] Lord C. Brugha T. S. Charman T. Cusack J. Dumas G. Frazier T.. (2020). Autism spectrum disorder. Nat. Rev. Dis. Primers 6, 5. doi: 10.1038/s41572-019-0138-4 31949163 PMC8900942

[B114] Loscher W. (2021). Single-target versus multi-target drugs versus combinations of drugs with multiple targets: preclinical and clinical evidence for the treatment or prevention of epilepsy. Front. Pharmacol. 12, 730257. doi: 10.3389/fphar.2021.730257 34776956 PMC8580162

[B115] Lukens J. R. Eyo U. B. (2022). Microglia and neurodevelopmental disorders. Annu. Rev. Neurosci. 45, 425–445. doi: 10.1146/annurev-neuro-110920-023056 35436413 PMC10449242

[B116] Luo H. Wang J. Qiao C. Ma N. Liu D. Zhang W.. (2015). Pycnogenol attenuates atherosclerosis by regulating lipid metabolism through the TLR4-NF-kappaB pathway. Exp. Mol. Med. 47, e191. doi: 10.1038/emm.2015.74 26492950 PMC4673476

[B117] Luzzi R. Belcaro G. Zulli C. Cesarone M. R. Cornelli U. Dugall M.. (2011). Pycnogenol(R) supplementation improves cognitive function, attention and mental performance in students. Panminerva Med. 53, 75–82. 22108481

[B118] Lyons-Warren A. M. McCormack M. C. Holder J. L. Jr. (2022). Sensory processing phenotypes in phelan-mcDermid syndrome and SYNGAP1-related intellectual disability. Brain Sci. 12. doi: 10.3390/brainsci12020137 PMC8869824 35203901

[B119] Maenner M. J. Shaw K. A. Baio J.. (2020). Prevalence of autism spectrum disorder among children aged 8 years - autism and developmental disabilities monitoring network, 11 sites, United States, 2016. MMWR Surveill Summ 69, 1–12. doi: 10.15585/mmwr.ss6904a1 PMC7119644 32214087

[B120] Manrique P. Bolduc B. Walk S. T. van der Oost J. de Vos W. M. Young M. J.. (2016). Healthy human gut phageome. Proc. Natl. Acad. Sci. U S A 113, 10400–10405. doi: 10.1073/pnas.1601060113 27573828 PMC5027468

[B121] Martin R. Rios-Covian D. Huillet E. Auger S. Khazaal S. Bermudez-Humaran L. G.. (2023). Faecalibacterium: a bacterial genus with promising human health applications. FEMS Microbiol Rev. 47. doi: 10.1093/femsre/fuad039 PMC10410495 37451743

[B122] Mittleman B. B. Castellanos F. X. Jacobsen L. K. Rapoport J. L. Swedo S. E. Shearer G. M.. (1997). Cerebrospinal fluid cytokines in pediatric neuropsychiatric disease. J. Immunol. 159, 2994–2999. doi: 10.4049/jimmunol.159.6.2994 9300724

[B123] Modi M. E. Sahin M. (2020). Tau: A novel entry point for mTOR-based treatments in autism spectrum disorder? Neuron 106, 359–361. doi: 10.1016/j.neuron.2020.04.019 32380047

[B124] Morris-Rosendahl D. J. Crocq M. A. (2020). Neurodevelopmental disorders-the history and future of a diagnostic concept. Dialogues Clin. Neurosci. 22, 65–72. doi: 10.31887/DCNS.2020.22.1/macrocq 32699506 PMC7365295

[B125] Mousavi S. M. Younesian S. Ejtahed H. S. (2025). The alteration of gut microbiota composition in patients with epilepsy: A systematic review and meta-analysis. Microb Pathog 199, 107266. doi: 10.1016/j.micpath.2024.107266 39736340

[B126] Mousavinejad E. Ghaffari M. A. Riahi F. Hajmohammadi M. Tiznobeyk Z. Mousavinejad M.. (2018). Coenzyme Q(10) supplementation reduces oxidative stress and decreases antioxidant enzyme activity in children with autism spectrum disorders. Psychiatry Res. 265, 62–69. doi: 10.1016/j.psychres.2018.03.061 29684771

[B127] Naderali E. Khaki A. A. Rad J. S. Ali-Hemmati A. Rahmati M. Charoudeh H. N.. (2018). Regulation and modulation of PTEN activity. Mol. Biol. Rep. 45, 2869–2881. doi: 10.1007/s11033-018-4321-6 30145641

[B128] Nattagh-Eshtivani E. Gheflati A. Barghchi H. Rahbarinejad P. Hachem K. Shalaby M. N.. (2022). The role of Pycnogenol in the control of inflammation and oxidative stress in chronic diseases: Molecular aspects. Phytother. Res. 36, 2352–2374. doi: 10.1002/ptr.v36.6 35583807

[B129] Nicolini C. Ahn Y. Michalski B. Rho J. M. Fahnestock M.. (2015). Decreased mTOR signaling pathway in human idiopathic autism and in rats exposed to valproic acid. Acta Neuropathol. Commun. 3, 3. doi: 10.1186/s40478-015-0184-4 25627160 PMC4307681

[B130] Nigam M. Mishra A. P. Deb V. K. Dimri D. B. Tiwari V. Bungau S. G.. (2023). Evaluation of the association of chronic inflammation and cancer: Insights and implications. BioMed. Pharmacother. 164, 115015. doi: 10.1016/j.biopha.2023.115015 37321055

[B131] Niwano Y. Kohzaki H. Shirato M. Shishido S. Nakamura K.. (2022). Metabolic fate of orally ingested proanthocyanidins through the digestive tract. Antioxidants (Basel) 12. doi: 10.3390/antiox12010017 PMC9854439 36670878

[B132] Packer L. Rimbach G. Virgili F. (1999). Antioxidant activity and biologic properties of a procyanidin-rich extract from pine (Pinus maritima) bark, pycnogenol. Free Radic. Biol. Med. 27, 704–724. doi: 10.1016/S0891-5849(99)00090-8 10490291

[B133] Pak S. W. Lee S. J. Kim W. I. Yang Y. G. Cho Y. K. Kim J. S.. (2024). The effects of Pycnogenol, a pine bark extract on pulmonary inflammation by Asian sand dust in mice. Vet Med. (Praha) 69, 8–17. doi: 10.17221/77/2023-VETMED 38465002 PMC10919100

[B134] Palmer E. E. Schofield D. Shrestha R. Kandula T. Macintosh R. Lawson J. A.. (2018). Integrating exome sequencing into a diagnostic pathway for epileptic encephalopathy: Evidence of clinical utility and cost effectiveness. Mol. Genet. Genomic Med. 6, 186–199. doi: 10.1002/mgg3.2018.6.issue-2 29314763 PMC5902395

[B135] Parihar R. Ganesh S. (2013). The SCN1A gene variants and epileptic encephalopathies. J. Hum. Genet. 58, 573–580. doi: 10.1038/jhg.2013.77 23884151

[B136] Partty A. Kalliomaki M. Wacklin P. Salminen S. Isolauri E.. (2015). A possible link between early probiotic intervention and the risk of neuropsychiatric disorders later in childhood: a randomized trial. Pediatr. Res. 77, 823–828. doi: 10.1038/pr.2015.51 25760553

[B137] Pearson-Smith J. N. Patel M. (2017). Metabolic dysfunction and oxidative stress in epilepsy. Int. J. Mol. Sci. 18. doi: 10.3390/ijms18112365 PMC5713334 29117123

[B138] Peng Q. L. Buz’Zard A. R. Lau B. H. (2002). Pycnogenol protects neurons from amyloid-beta peptide-induced apoptosis. Brain Res. Mol. Brain Res. 104, 55–65. doi: 10.1016/S0169-328X(02)00263-2 12117551

[B139] Peng Y. J. Lee C. H. Wang C. C. Salter D. M. Lee H. S.. (2012). Pycnogenol attenuates the inflammatory and nitrosative stress on joint inflammation induced by urate crystals. Free Radic. Biol. Med. 52, 765–774. doi: 10.1016/j.freeradbiomed.2011.12.003 22198264

[B140] Perez-Burillo S. Navajas-Porras B. Lopez-Maldonado A. Hinojosa-Nogueira D. Pastoriza S. Rufian-Henares J. A.. (2021). Green tea and its relation to human gut microbiome. Molecules 26. doi: 10.3390/molecules26133907 PMC8271705 34206736

[B141] Peterson T. R. Sengupta S. S. Harris T. E. Carmack A. E. Kang S. A. Balderas E.. (2011). mTOR complex 1 regulates lipin 1 localization to control the SREBP pathway. Cell 146, 408–420. doi: 10.1016/j.cell.2011.06.034 21816276 PMC3336367

[B142] Prehn-Kristensen A. Zimmermann A. Tittmann L. Lieb W. Schreiber S. Baving L.. (2018). Reduced microbiome alpha diversity in young patients with ADHD. PloS One 13, e0200728. doi: 10.1371/journal.pone.0200728 30001426 PMC6042771

[B143] Qiu Y. Song B. Xie M. Tao Y. Yin Z. Wang M.. (2024). Causal links between gut microbiomes, cytokines and risk of different subtypes of epilepsy: a Mendelian randomization study. Front. Neurosci. 18, 1397430. doi: 10.3389/fnins.2024.1397430 38855442 PMC11157073

[B144] Rafiyan M. Sadeghmousavi S. Akbarzadeh M. Rezaei N.. (2023). Experimental animal models of chronic inflammation. Curr. Res. Immunol. 4, 100063. doi: 10.1016/j.crimmu.2023.100063 37334102 PMC10276141

[B145] Retuerto M. Al-Shakhshir H. Herrada J. McCormick T. S. Ghannoum M. A.. (2024). Analysis of gut bacterial and fungal microbiota in children with autism spectrum disorder and their non-autistic siblings. Nutrients 16. doi: 10.3390/nu16173004 PMC11396985 39275319

[B146] Robinson-Agramonte M. L. A. Noris Garcia E. Fraga Guerra J. Vega Hurtado Y. Antonucci N. Semprun-Hernandez N.. (2022). Immune dysregulation in autism spectrum disorder: what do we know about it? Int. J. Mol. Sci. 23. doi: 10.3390/ijms23063033 PMC8955336 35328471

[B147] Rodriguez J. M. Murphy K. Stanton C. Ross R. P. Kober O. I. Juge N.. (2015). The composition of the gut microbiota throughout life, with an emphasis on early life. Microb Ecol. Health Dis. 26, 26050. doi: 10.3402/mehd.v26.26050 25651996 PMC4315782

[B148] Rohdewald P. (2002). A review of the French maritime pine bark extract (Pycnogenol), a herbal medication with a diverse clinical pharmacology. Int. J. Clin. Pharmacol. Ther. 40, 158–168. doi: 10.5414/CPP40158 11996210

[B149] Rohdewald P. J. (2018). Review on sustained relief of osteoarthritis symptoms with a proprietary extract from pine bark, pycnogenol. J. Med. Food 21, 1–4. doi: 10.1089/jmf.2017.0015 28836883 PMC5775113

[B150] Safak B. Altunan B. Topcu B. Eren Topkaya A.. (2020). The gut microbiome in epilepsy. Microb Pathog 139, 103853. doi: 10.1016/j.micpath.2019.103853 31730997

[B151] Sandin S. Lichtenstein P. Kuja-Halkola R. Hultman C. Larsson H. Reichenberg A.. (2017). The heritability of autism spectrum disorder. JAMA 318, 1182–1184. doi: 10.1001/jama.2017.12141 28973605 PMC5818813

[B152] Schantz M. Erk T. Richling E. (2010). Metabolism of green tea catechins by the human small intestine. Biotechnol. J. 5, 1050–1059. doi: 10.1002/biot.201000214 20931601

[B153] Schneider K. M. Blank N. Alvarez Y. Thum K. Lundgren P. Litichevskiy L.. (2023). The enteric nervous system relays psychological stress to intestinal inflammation. Cell 186, 2823–2838. doi: 10.1016/j.cell.2023.05.001 37236193 PMC10330875

[B154] Schnorr I. Siegl A. Luckhardt S. Wenz S. Friedrichsen H. El Jomaa H.. (2024). Inflammatory biotype of ADHD is linked to chronic stress: a data-driven analysis of the inflammatory proteome. Transl. Psychiatry 14, 37. doi: 10.1038/s41398-023-02729-3 38238292 PMC10796401

[B155] Schoonees A. Visser J. Musekiwa A. Volmink J.. (2012). Pycnogenol((R)) for the treatment of chronic disorders. Cochrane Database Syst. Rev. 2, CD008294. doi: 10.1002/14651858.CD008294.pub3 22513958

[B156] Schwartzer J. J. Careaga M. Coburn M. A. Rose D. R. Hughes H. K. Ashwood P.. (2017). Behavioral impact of maternal allergic-asthma in two genetically distinct mouse strains. Brain Behav. Immun. 63, 99–107. doi: 10.1016/j.bbi.2016.09.007 27622677 PMC5346064

[B157] Selkrig J. Wong P. Zhang X. Pettersson S.. (2014). Metabolic tinkering by the gut microbiome: Implications for brain development and function. Gut Microbes 5, 369–380. doi: 10.4161/gmic.28681 24685620 PMC4153776

[B158] Sensi S. L. Granzotto A. Siotto M. Squitti R.. (2018). Copper and zinc dysregulation in alzheimer’s disease. Trends Pharmacol. Sci. 39, 1049–1063. doi: 10.1016/j.tips.2018.10.001 30352697

[B159] Shaaban S. Y. El Gendy Y. G. Mehanna N. S. El-Senousy W. M. El-Feki H. S. A. Saad K.. (2018). The role of probiotics in children with autism spectrum disorder: A prospective, open-label study. Nutr. Neurosci. 21, 676–681. doi: 10.1080/1028415X.2017.1347746 28686541

[B160] Sharma A. Mehan S. (2021). Targeting PI3K-AKT/mTOR signaling in the prevention of autism. Neurochem. Int. 147, 105067. doi: 10.1016/j.neuint.2021.105067 33992742

[B161] Singh V. Roth S. Llovera G. Sadler R. Garzetti D. Stecher B.. (2016). Microbiota dysbiosis controls the neuroinflammatory response after stroke. J. Neurosci. 36, 7428–7440. doi: 10.1523/JNEUROSCI.1114-16.2016 27413153 PMC6705544

[B162] Singh A. Upadhayay S. Mehan S. (2021). Understanding abnormal c-JNK/p38MAPK signaling overactivation involved in the progression of multiple sclerosis: possible therapeutic targets and impact on neurodegenerative diseases. Neurotox Res. 39, 1630–1650. doi: 10.1007/s12640-021-00401-6 34432262

[B163] Sinn N. (2008). Nutritional and dietary influences on attention deficit hyperactivity disorder. Nutr. Rev. 66, 558–568. doi: 10.1111/j.1753-4887.2008.00107.x 18826452

[B164] Sivamaruthi B. S. Suganthy N. Kesika P. Chaiyasut C.. (2020). The role of microbiome, dietary supplements, and probiotics in autism spectrum disorder. Int. J. Environ. Res. Public Health 17. doi: 10.3390/ijerph17082647 PMC7215504 32290635

[B165] Solleiro-Villavicencio H. Rivas-Arancibia S. (2018). Effect of chronic oxidative stress on neuroinflammatory response mediated by CD4(+)T cells in neurodegenerative diseases. Front. Cell Neurosci. 12, 114. doi: 10.3389/fncel.2018.00114 29755324 PMC5934485

[B166] Sun B. Wang Y. Bai J. Li X. Ma L. Man S.. (2024). Litchi procyanidins ameliorate DSS-induced colitis through gut microbiota-dependent regulation of treg/th17 balance. J. Agric. Food Chem. 72, 24823–24832. doi: 10.1021/acs.jafc.4c05577 39315595

[B167] Szopinska-Tokov J. Dam S. Naaijen J. Konstanti P. Rommelse N. Belzer C.. (2020). Investigating the gut microbiota composition of individuals with attention-deficit/hyperactivity disorder and association with symptoms. Microorganisms 8. doi: 10.3390/microorganisms8030406 PMC7143990 32183143

[B168] Takata A. Nakashima M. Saitsu H. Mizuguchi T. Mitsuhashi S. Takahashi Y.. (2019). Comprehensive analysis of coding variants highlights genetic complexity in developmental and epileptic encephalopathy. Nat. Commun. 10, 2506. doi: 10.1038/s41467-019-10482-9 31175295 PMC6555845

[B169] Tenenbaum S. Paull J. C. Sparrow E. P. Dodd D. K. Green L.. (2002). An experimental comparison of Pycnogenol and methylphenidate in adults with Attention-Deficit/Hyperactivity Disorder (ADHD). J. Atten Disord. 6, 49–60. doi: 10.1177/108705470200600201 12142861

[B170] Tengeler A. C. Dam S. A. Wiesmann M. Naaijen J. van Bodegom M. Belzer C.. (2020). Gut microbiota from persons with attention-deficit/hyperactivity disorder affects the brain in mice. Microbiome 8, 44. doi: 10.1186/s40168-020-00816-x 32238191 PMC7114819

[B171] Tomova A. Husarova V. Lakatosova S. Bakos J. Vlkova B. Babinska K.. (2015). Gastrointestinal microbiota in children with autism in Slovakia. Physiol. Behav. 138, 179–187. doi: 10.1016/j.physbeh.2014.10.033 25446201

[B172] Treangen T. J. Wagner J. Burns M. P. Villapol S.. (2018). Traumatic brain injury in mice induces acute bacterial dysbiosis within the fecal microbiome. Front. Immunol. 9, 2757. doi: 10.3389/fimmu.2018.02757 30546361 PMC6278748

[B173] Trebaticka J. Durackova Z. (2015). Psychiatric disorders and polyphenols: can they be helpful in therapy? Oxid. Med. Cell Longev 2015, 248529. doi: 10.1155/2015/248529 26180581 PMC4477218

[B174] Trebaticka J. Kopasova S. Hradecna Z. Cinovsky K. Skodacek I. Suba J.. (2006). Treatment of ADHD with French maritime pine bark extract, Pycnogenol. Eur. Child Adolesc. Psychiatry 15, 329–335. doi: 10.1007/s00787-006-0538-3 16699814

[B175] Treiman D. M. (2001). GABAergic mechanisms in epilepsy. Epilepsia 42 Suppl 3, 8–12. doi: 10.1046/j.1528-1157.2001.042suppl.3008.x 11520315

[B176] Trovato F. Parra R. Pracucci E. Landi S. Cozzolino O. Nardi G.. (2020). Modelling genetic mosaicism of neurodevelopmental disorders *in vivo* by a Cre-amplifying fluorescent reporter. Nat. Commun. 11, 6194. doi: 10.1038/s41467-020-19864-w 33273479 PMC7713426

[B177] Tural Hesapcioglu S. Kasak M. Citak Kurt A. N. Ceylan M. F.. (2017). High monocyte level and low lymphocyte to monocyte ratio in autism spectrum disorders. Int. J. Dev. Disabil. 65, 73–81. doi: 10.1080/20473869.2017.1371369 34141326 PMC8115457

[B178] Turner T. J. Zourray C. Schorge S. Lignani G.. (2021). Recent advances in gene therapy for neurodevelopmental disorders with epilepsy. J. Neurochem. 157, 229–262. doi: 10.1111/jnc.v157.2 32880951 PMC8436749

[B179] Uhlenhut K. Hogger P. (2012). Facilitated cellular uptake and suppression of inducible nitric oxide synthase by a metabolite of maritime pine bark extract (Pycnogenol). Free Radic. Biol. Med. 53, 305–313. doi: 10.1016/j.freeradbiomed.2012.04.013 22569413

[B180] Ullah H. Arbab S. Tian Y. Liu C. Q. Chen Y. Qijie L.. (2023). The gut microbiota-brain axis in neurological disorder. Front. Neurosci. 17, 1225875. doi: 10.3389/fnins.2023.1225875 37600019 PMC10436500

[B181] Ullah H. Arbab S. Tian Y. Chen Y. Liu C. Q. Li Q.. (2024). Crosstalk between gut microbiota and host immune system and its response to traumatic injury. Front. Immunol. 15, 1413485. doi: 10.3389/fimmu.2024.1413485 39144142 PMC11321976

[B182] Ullah H. Arbab S. Chang C. Bibi S. Muhammad N. Rehman S. U.. (2025). Gut microbiota therapy in gastrointestinal diseases. Front. Cell Dev. Biol. 13, 1514636. doi: 10.3389/fcell.2025.1514636 40078367 PMC11897527

[B183] Usui N. Iwata K. Miyachi T. Takagai S. Wakusawa K. Nara T.. (2020). VLDL-specific increases of fatty acids in autism spectrum disorder correlate with social interaction. EBioMedicine 58, 102917. doi: 10.1016/j.ebiom.2020.102917 32739868 PMC7393524

[B184] van der Pol A. van Gilst W. H. Voors A. A. van der Meer P.. (2019). Treating oxidative stress in heart failure: past, present and future. Eur. J. Heart Fail 21, 425–435. doi: 10.1002/ejhf.2019.21.issue-4 30338885 PMC6607515

[B185] Vazquez J. C. Martin de la Torre O. Lopez Palome J. Redolar-Ripoll D.. (2022). Effects of caffeine consumption on attention deficit hyperactivity disorder (ADHD) treatment: A systematic review of animal studies. Nutrients 14. doi: 10.3390/nu14040739 PMC8875377 35215389

[B186] Verlaet A. A. Ceulemans B. Verhelst H. Van West D. De Bruyne T. Pieters L.. (2017). Effect of Pycnogenol(R) on attention-deficit hyperactivity disorder (ADHD): study protocol for a randomised controlled trial. Trials 18, 145. doi: 10.1186/s13063-017-1879-6 28351412 PMC5370458

[B187] Visternicu M. Rarinca V. Burlui V. Halitchi G. Ciobica A. Singeap A. M.. (2024). Investigating the impact of nutrition and oxidative stress on attention deficit hyperactivity disorder. Nutrients 16. doi: 10.3390/nu16183113 PMC11435085 39339712

[B188] Vuong H. E. Pronovost G. N. Williams D. W. Coley E. J. L. Siegler E. L. Qiu A.. (2020). The maternal microbiome modulates fetal neurodevelopment in mice. Nature 586, 281–286. doi: 10.1038/s41586-020-2745-3 32968276 PMC7554197

[B189] Vuong H. E. Hsiao E. Y. (2017). Emerging roles for the gut microbiome in autism spectrum disorder. Biol. Psychiatry 81, 411–423. doi: 10.1016/j.biopsych.2016.08.024 27773355 PMC5285286

[B190] Waldbaum S. Liang L. P. Patel M. (2010). Persistent impairment of mitochondrial and tissue redox status during lithium-pilocarpine-induced epileptogenesis. J. Neurochem. 115, 1172–1182. doi: 10.1111/j.1471-4159.2010.07013.x 21219330 PMC4878708

[B191] Wan L. Ge W. R. Zhang S. Sun Y. L. Wang B. Yang G.. (2020). Case-control study of the effects of gut microbiota composition on neurotransmitter metabolic pathways in children with attention deficit hyperactivity disorder. Front. Neurosci. 14, 127. doi: 10.3389/fnins.2020.00127 32132899 PMC7040164

[B192] Wang L. Wang B. Wu C. Wang J. Sun M.. (2023). Autism spectrum disorder: neurodevelopmental risk factors, biological mechanism, and precision therapy. Int. J. Mol. Sci. 24. doi: 10.3390/ijms24031819 PMC9915249 36768153

[B193] Wang Q. Yang Q. Liu X. (2023). The microbiota-gut-brain axis and neurodevelopmental disorders. Protein Cell 14, 762–775. doi: 10.1093/procel/pwad026 37166201 PMC10599644

[B194] Wei F. Yan L. M. Su T. He N. Lin Z. J. Wang J.. (2017). Ion channel genes and epilepsy: functional alteration, pathogenic potential, and mechanism of epilepsy. Neurosci. Bull. 33, 455–477. doi: 10.1007/s12264-017-0134-1 28488083 PMC5567559

[B195] Weichmann F. Rohdewald P. (2024). Pycnogenol((R)) French maritime pine bark extract in randomized, double-blind, placebo-controlled human clinical studies. Front. Nutr. 11, 1389374. doi: 10.3389/fnut.2024.1389374 38757130 PMC11096518

[B196] Weng M. Xie X. Liu C. Lim K. L. Zhang C. W. Li L.. (2018). The sources of reactive oxygen species and its possible role in the pathogenesis of parkinson’s disease. Parkinsons Dis. 2018, 9163040. doi: 10.1155/2018/9163040 30245802 PMC6139203

[B197] Wu H. Jiawei X. Wen Z. Han Y. Liu Y. Chen S.. (2025). Microbiome-gut-brain profiles in schizophrenia and their potential link to cognitive performance: findings from a case-control study. Schizophr. Bull. doi: 10.1093/schbul/sbaf028 PMC12597491 40131173

[B198] Xia R. Ji C. Zhang L. (2017). Neuroprotective effects of pycnogenol against oxygen-glucose deprivation/reoxygenation-induced injury in primary rat astrocytes via NF-kappaB and ERK1/2 MAPK pathways. Cell Physiol. Biochem. 42, 987–998. doi: 10.1159/000478681 28662519

[B199] Yan B. Sun Y. Zeng J. Chen Y. Li C. Song P.. (2019). Combined use of vitamin E and nimodipine ameliorates dibutyl phthalate-induced memory deficit and apoptosis in mice by inhibiting the ERK 1/2 pathway. Toxicol. Appl. Pharmacol. 368, 1–17. doi: 10.1016/j.taap.2019.02.008 30776390

[B200] Yang T. Chen L. Dai Y. Jia F. Hao Y. Li L.. (2022). Vitamin A status is more commonly associated with symptoms and neurodevelopment in boys with autism spectrum disorders-A multicenter study in China. Front. Nutr. 9, 851980. doi: 10.3389/fnut.2022.851980 35495950 PMC9038535

[B201] Zeng Y. Cao S. Yang H. (2023). Roles of gut microbiome in epilepsy risk: A Mendelian randomization study. Front. Microbiol 14, 1115014. doi: 10.3389/fmicb.2023.1115014 36922970 PMC10010438

[B202] Zhang H. Wei M. Sun Q. Yang T. Lu X. Feng X.. (2020). Lycopene ameliorates chronic stress-induced hippocampal injury and subsequent learning and memory dysfunction through inhibiting ROS/JNK signaling pathway in rats. Food Chem. Toxicol. 145, 111688. doi: 10.1016/j.fct.2020.111688 32810585

[B203] Zhao C. Tang J. Li X. Yan Z. Zhao L. Lang W.. (2022). Beneficial effects of procyanidin B2 on adriamycin-induced nephrotic syndrome mice: the multi-action mechanism for ameliorating glomerular permselectivity injury. Food Funct. 13, 8436–8464. doi: 10.1039/D1FO03616E 35861207

[B204] Zheng Y. Chen J. (2024). Voltage-gated potassium channels and genetic epilepsy. Front. Neurol. 15, 1466075. doi: 10.3389/fneur.2024.1466075 39434833 PMC11492950

[B205] Zhou Y. Kaiser T. Monteiro P. Zhang X. van der Goes M. S. Wang D.. (2016). Mice with shank3 mutations associated with ASD and schizophrenia display both shared and distinct defects. Neuron 89, 147–162. doi: 10.1016/j.neuron.2015.11.023 26687841 PMC4754122

